# Expanding and Enhancing Neural Network-Based QM-AI Models for the Accurate Prediction of Halogen‑π Interaction Energies in Protein Contexts

**DOI:** 10.1021/acs.jcim.5c03249

**Published:** 2026-04-14

**Authors:** Marc U. Engelhardt, Finn Mier, Markus O. Zimmermann, Frank M. Boeckler

**Affiliations:** † Laboratory for Molecular Design & Pharmaceutical Biophysics, Institute of Pharmaceutical Sciences, Department of Pharmacy and Biochemistry, 9188 Eberhard Karls Universität Tübingen, Tübingen 72076, Germany; ‡ Interfaculty Institute for Biomedical Informatics (IBMI), Eberhard Karls Universität Tübingen, Tübingen 72076, Germany

## Abstract

In this study, we extend a previously introduced QM-AI strategy for predicting halogen···π interaction energies from a single aromatic model (representing phenylalanine) to multiple biologically relevant aromatic environments. Herein, neural network models were developed for halogen···π interactions involving phenol, imidazole, and indole, serving as model systems for the aromatic side chain residues of tyrosine, histidine, and tryptophan. Large, systematically generated datasets of halobenzene-aromatic system complexes (in total, over 18 million interaction geometries) were evaluated at the MP2/TZVPP level of theory and represented by compact geometric descriptors to train residue-specific neural networks. Across all systems, the models reproduce quantum-mechanical reference energies with high accuracy (R^2^ > 0.98 and RMSE < 0.5 kJ/mol) within the targeted σ-hole interaction domain and retain robust performance on independent, randomly generated geometry and PDB-derived test sets. Model limitations are primarily associated with geometric arrangements outside the training distribution, such as π···π, C–H···π, or other non-σ-hole interaction motifs. By augmenting the training data with additional randomly generated geometries, model robustness and generalization were further improved without modifying the underlying network architecture. Overall, this work establishes a scalable and transferable QM-AI strategy for the rapid and accurate prediction of halogen···π interaction energies across diverse aromatic environments, enabling near-quantum-mechanical accuracy at negligible computational cost and supporting future applications in structure-based drug design.

## Graphical abstract

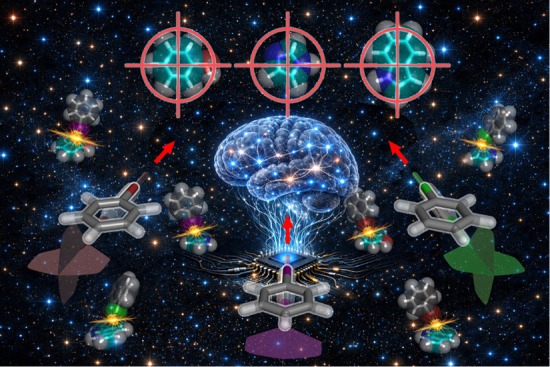

## Introduction

Understanding the individual contributions of different noncovalent interactions is crucial for elucidating biomolecular functions and guiding the rational design of potential therapeutics.^1–5^ Halogen bonding (XB) is a directional noncovalent interaction between a halogen atom acting as an electrophilic site due to its σ-hole, a positive electrostatic region along the extension of the C-X bond (X = Cl, Br, or I), and a nucleophilic site, such as a lone pair or π-system.^6–10^ The highly anisotropic electron distribution around the halogen atoms results in a pronounced lateral electron density oriented perpendicular to the R-X bond axis. When the substituent R exerts a pronounced electron-withdrawing influence on the halogen (X), such tuning effects lead to a substantial increase in halogen-bond strength.^11–16^ In biomolecular systems, halogen bonds have been increasingly recognized as relevant contributors to protein–ligand binding and drug-target recognition.^17–27^ Aromatic π-systems, heteroaromatic residues, and backbone carbonyl groups can act as halogen bond acceptors, giving rise to well-defined interaction motifs in protein–ligand complexes. Consequently, halogen substitution has become an established strategy in medicinal chemistry to modulate binding affinity, selectivity, and pharmacokinetic properties.^28–35^

Computational chemistry has played a central role in elucidating both the structural preferences and energetic characteristics of halogen bond interactions.^36–38^ Previous studies have focused predominantly on halogen bonds formed with classical nucleophilic sites commonly found in protein-binding pockets, such as backbone carbonyl oxygen atoms,^39,40^ peptide π-surfaces,^41^ heteroatoms in amino acid side chains,^42,43^ carboxylate and carboxamide functionalities,^44^ and coordinated or structural water molecules.^45,46^

Recently, we investigated halogen bonding to the electron-rich π-system of the aromatic amino acid side chain of phenylalanine using high-level quantum-mechanical calculations. We demonstrated that MP2 calculations with sufficiently large basis sets (TZVPP) reproduce CCSD­(T)/CBS reference interaction energies for halogen···π complexes with high accuracy, while reducing computational cost by approximately 2 orders of magnitude relative to CCSD­(T).^47^ This efficiency gain enabled the generation of large, high-quality reference datasets suitable for systematic analysis. Building on this foundation, we subsequently trained neural network models on the MP2-derived data to predict halogen···π interaction energies directly from geometric descriptors.^48^ These models achieved excellent predictive accuracy at negligible computational cost, corresponding to an overall acceleration of roughly 10 orders of magnitude compared to CCSD­(T) calculations. The results demonstrated that directional σ-hole interactions can be reliably captured using well-chosen geometric features by exploiting a methodological and hierarchical “double-jump” strategy from CCSD­(T) to MP2 and ultimately to neural networks, providing a practical alternative to explicit quantum-mechanical evaluations of halogen···π interactions.

In the present study, we build directly on this framework and extend it beyond a single aromatic reference system, implementing aromatic environments representative of the remaining amino acid side chains. Specifically, we investigate halogen···π interactions involving phenol, imidazole, and indole, serving as model systems for the aromatic side chain residues of tyrosine, histidine, and tryptophan, respectively. For histidine, only the neutral imidazole form was considered, while the protonated imidazolinium state was excluded. Although histidine can be protonated in certain protein environments (e.g., kinase active sites), explicitly accounting for variable protonation states would substantially increase the model complexity and require extensive site-specific preprocessing. Moreover, protonated histidine (imidazolinium) and related cationic π-systems, such as the guanidinium group of arginine, represent a distinct class of positively charged aromatic interaction motifs that are beyond the scope of the present work. From a physicochemical perspective, positively charged histidine is also expected to be less favorable for σ-hole-driven halogen bonding, as electrostatic attraction would preferentially promote charge-assisted hydrogen bonding or interactions with the lateral electron-rich belt of the halogen rather than directional σ-hole contacts, potentially leading to reoriented binding geometries. In contrast, neutral imidazole provides a more plausible acceptor environment for stabilizing genuine halogen···π interactions. A systematic treatment of positively charged π-systems, including both imidazolinium and guanidinium motifs, therefore represents a related and promising direction for future investigation. For each system, large and systematically generated datasets of approximately 6 million interaction geometries between halobenzenes (chlorobenzene, bromobenzene, and iodobenzene) and the corresponding aromatic acceptor were constructed and evaluated at the MP2/TZVPP level of theory. As in our previous work, the interaction geometries were sampled on structured grids defined by halogen···π-plane distances (from *d*_min(X···π‑plane)_ = 2.75 Å to *d*_max(X···π‑plane)_ = 4.50 Å) and angular constraints (maximum deviation of 40° between the C-X bond vector and the π-plane normal) chosen to selectively represent directional σ-hole interactions while minimizing contributions from competing motifs such as π···π or C–H···π contacts. The resulting quantum-mechanical reference data were used to train dedicated neural network models for each aromatic system in a supervised learning approach. Model performance was assessed using both test sets of randomly generated geometries and geometries derived from protein crystal structures of the Protein Data Bank (PDB),^49^ enabling a direct evaluation of transferability to biologically relevant examples. Here again, only neutral histidine/imidazole motifs were retained, as reliable inclusion of protonated histidine would require prior protonation-state assignment, binding-site environment analysis, and optimization of hydrogen-bond networks for each structure, which is computationally prohibitive on the scale considered in this study.

In addition to extending the framework to multiple aromatic side-chain models, the present study explicitly examines the modular expandability of the trained neural network models. A central premise of the approach is that model robustness and transferability can be systematically improved by incorporating additional training data without modifying the underlying network architecture. To assess this capability, we further generated approximately 100,000 randomly sampled interaction geometries for each aromatic system and halogen type and employed them in a retraining step. These augmented datasets were designed to broaden the sampled feature space beyond the structured grids used for the initial training, thereby enabling a targeted evaluation of how expanded training distributions influence predictive performance, generalization to previously challenging geometries, and transferability to PDB-derived interaction motifs. By combining systematic data generation, controlled retraining, and evaluation on both randomly sampled and biologically derived test sets, this study aims to establish a scalable and adaptable machine-learning framework for the efficient prediction of halogen···π interaction energies across diverse aromatic environments. It should be noted that the present models predict gas-phase interaction energies (Δ*E*) rather than binding free energies (Δ*G*). In practical structure-based drug design workflows, solvation, desolvation penalties, and entropic contributions are accounted for by the surrounding docking or scoring framework, while the present models provide an interaction-specific description of directional halogen···π interactions. This represents a further step toward integration into structure-based drug design workflows, including molecular docking and scoring applications.

## Results and Discussion

### Model Training and Validation

First, we conducted single-point calculations of the generated interaction geometries. Energies were calculated by using the supermolecular approach (eq 1). Features were derived from the individual interaction geometries. A complete list of all features of the individual model systems, along with descriptions and a more detailed schematic of the feature definitions, is provided in the Supporting Information (Figure S1, Tables S1 and S2). To ensure the feature space is translationally and rotationally independent of the underlying coordinate system, we chose pairwise distances and angles. For the training process, the resulting datasets were partitioned into training, validation, and test subsets (for more details, see Materials and Methods). Hyperparameter search and training processes were performed in a stratified 5-fold cross-validation with an 80%/20% test/validation split. Final model training on the best-performing model configuration was conducted using a slightly adapted training subset (95% of the data), with evaluation on the corresponding validation subset (5% of the data) at the end of each epoch. Training was terminated upon satisfaction of the early stopping criterion. Model evaluation of the withheld test sets shows excellent accuracy across all three models, with only very few large energy deviations (ΔΔ*E*, eq 2). For each system, the vast majority of predictions fall within ±0.5 kJ/mol, and only a small fraction exceeds this range, with 1.49% for phenol, 5.04% for imidazole, and 2.02% for indole. For phenol, the maximum and minimum deviations were +4.08 and −3.11 kJ/mol, respectively, yielding an R_phenol_^2^ = 0.9983 and an RMSE_phenol‑test_ = 0.15 kJ/mol, demonstrating that predictive performance remains strong even on unseen data (Figure S2a/b).

Similarly, the imidazole model exhibited maximum and minimum deviations of +8.92 and −7.84 kJ/mol, respectively, achieving an R_imidazole_^2^ = 0.9919 and an RMSE_imidazole‑test_ = 0.28 kJ/mol (Figure S2c/d). For indole, the deviations ranged from +3.76 to −2.95 kJ/mol, with an R_indole_^2^ = 0.9982 and an RMSE_indole‑test_ = 0.17 kJ/mol (Figure S2e/f). Despite these promising results, the performance may be somewhat inflated due to similarities between the training and test sets, potentially introducing data leakage, as both likely occupy the same region of feature space. Therefore, a more rigorous assessment requires evaluating the models on entirely novel data that fall outside the distribution of the training set to truly estimate their generalization capabilities. In addition to such fully unseen, out-of-distribution samples, it is equally important to assess data points that lie within the training distribution but involve interpolated parameter combinations. This ensures that the models are not merely memorizing known values but can also generalize smoothly across the interior of the feature space. Therefore, a two-step evaluation scheme of the individual models’ test datasets was applied. In the first step (i), a subset containing only data points with features lying within the training distribution (2.75 Å ≤ d_X···π‑plane_ ≤ 4.5 Å; α_C‑X···⊥(π‑plane)_ ≤ 40°) was analyzed. In the second step (ii), distance and angle constraints were dropped, and the full test dataset was evaluated. This removal of the restrictions was intended to test and identify the models’ limitations and ability to generalize on unseen data on the edges or outside of the feature space covered by the underlying training distribution.

### Predicting Adduct Formation Energies of Unseen Datasets

To rigorously assess the generalization capability of the trained models, we generated independent test sets comprising interaction geometries with randomly sampled translational and rotational parameters for each molecular system. Each test set contained 60,000 structures (20,000 per halogen). Single-point interaction energies were computed at the MP2/TZVPP level of theory, and repulsive geometries with interaction energies exceeding +10 kJ/mol were discarded. The resulting curated datasets contained N_phenol_ = 56,591, N_imidazole_ = 57,297, and N_indol_ = 55,415 complexes. Geometric features were extracted from each complex. A detailed table incorporating the individual data points, energies *E*_calc_ and *E*_pred_, as well as the difference between both, is provided in spreadsheet format in the Supporting Information. To examine the dependence of model performance on the amount of training data, we constructed learning curves by training the models on progressively larger fractions of the original training set. Model accuracy was evaluated both on the internal test split and on the independent random-geometry test set described above. A detailed representation of the learning curves is provided in the Supporting Information (Figure S7). In both evaluations, the prediction errors decrease rapidly with increasing training set size and approach a plateau once approximately 30–40% of the data is included. Beyond this point, further enlargement of the training set yields only marginal improvements in MAE and RMSE. These results indicate that the chosen feature representation efficiently captures the relevant geometry–energy relationships, while the full dataset primarily contributes to improved robustness and coverage of the interaction space.

### Phenol Model Evaluation

The halobenzene···phenol model was evaluated using the two-step protocol described above. In the constrained subset (step i, N = 16,062), which contains only geometries lying within the feature space sampled during training (2.75 Å ≤ d_X···π‑plane_ ≤ 4.5 Å, α_C‑X···⊥(π‑plane)_ ≤ 40°), the model attained excellent accuracy (R^2^ = 0.9935, RMSE = 0.27 kJ/mol), essentially matching the performance observed during training with low energy deviations between ΔΔ*E* = +3.21 and −3.65 kJ/mol, and 5.51% lying beyond ±0.5 kJ/mol (Figure S3).

Evaluating the model on the full dataset (step ii, N = 56,591), where all geometric constraints were removed, resulted in a modest decrease in accuracy but still strong predictive ability (R^2^ = 0.9644, RMSE = 0.90 kJ/mol). The observed deviations ranged from ΔΔ*E* = +27.98 to −16.58 kJ/mol, with 12.41% lying beyond ±0.5 kJ/mol. To better understand the origins of these deviations, a parity plot of *E*_calc_ and *E*_pred_ was generated and colored by Mahalanobis distance^50^ (MD) (Figure 1). The MD reflects how far each geometry lies from the center of the training feature distribution, accounting for feature correlations through the covariance matrix (eq 3). Most points lie close to the diagonal and exhibit low MDs, indicating close agreement between predicted and reference energies for structures similar to those encountered during training. In contrast, points with larger errors typically show elevated MDs, demonstrating a clear link between reduced predictive performance and increasing dissimilarity from the training distribution. Four representative outliers (A–D) were selected for closer inspection (Figure 2). It should be noted that these subsequently discussed examples are some of the very few strong outliers, highlighting “worst-case” scenarios. Calculated and predicted adduct formation energies, energy differences, distances d_X···π‑plane_ between the halogen and the π-plane, as well as the angle between the C-X vector and the normal to the π-plane α_C‑X···⊥(π‑plane)_ of these phenol outliers are listed in Table 1. Outliers can arise for two principal reasons: (a) the geometry resides outside the feature space spanned by the training data, requiring the model to extrapolate; or (b) the geometry lies within the training distribution but is still poorly predicted, implying limitations in the model architecture or training strategy.

**Figure 1:**
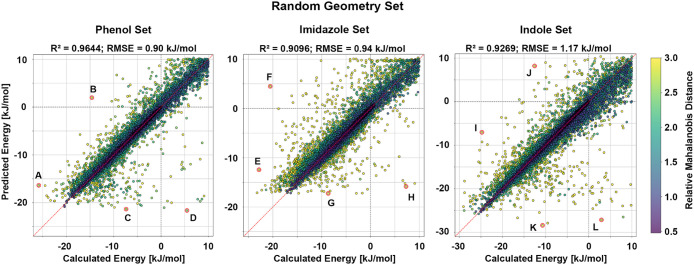
Model performance on the random geometry set. Calculated adduct formation energies are plotted against the corresponding predicted values for the phenol, imidazole, and indole models. The phenol model achieves an R^2^ = 0.9644 with an RMSE = 0.90 kJ/mol, the imidazole model achieves an R^2^ = 0.9096 with an RMSE = 0.94 kJ/mol, and the indole model achieves an R^2^ = 0.9269 with an RMSE = 1.17 kJ/mol. The red dashed line denotes perfect agreement between the calculated and predicted energies, while the gray dashed lines mark the transition between negative and positive energies. Data points are colored according to their relative MD, as indicated by the color scale. Data points highlighted by red circles and labeled A–L correspond to selected outliers and are discussed in detail in Figure 2.

**Figure 2:**
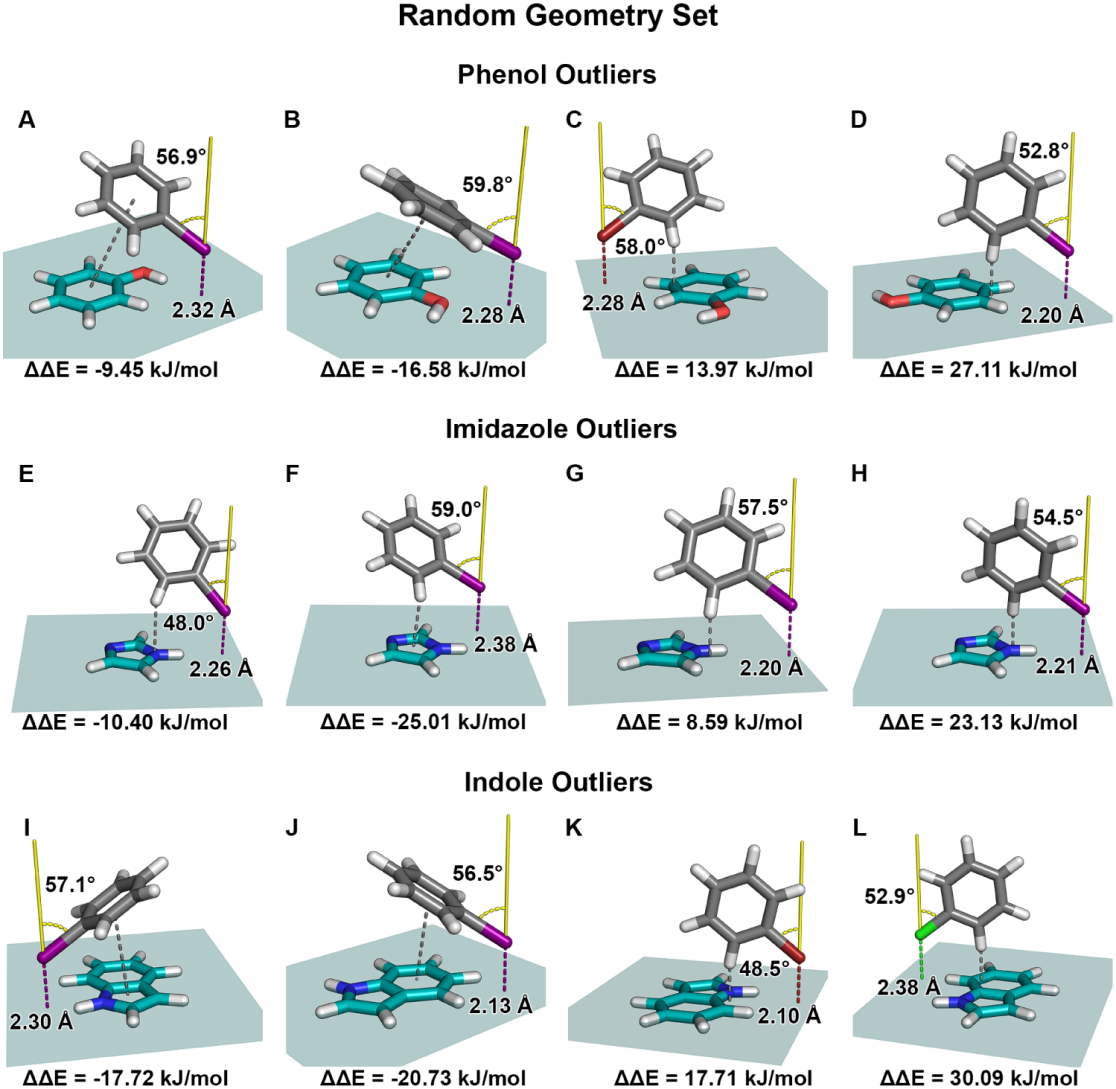
Interaction geometries for selected outliers from the random geometry set involving phenol, imidazole, and indole, as identified in the scatter plot in Figure 1. These structures correspond to data points exhibiting large deviations between calculated and predicted adduct formation energies. Geometries A–D depict the relative orientation of the halobenzene (gray) with respect to the phenol π-system (teal). Shown are the halogen distance d_X···π‑plane_ (dashed line, colored according to the halogen) to the π-system plane in Å, the torsion angle α_C‑X···⊥(π‑plane)_ (yellow) between the C-X bond vector and the normal to the π-system plane in degrees, and the corresponding energy difference ΔΔ*E*. The gray dashed line indicates the type of interaction that may contribute to the observed prediction error. For clarity, the teal plane also illustrates the extent of the training grid used in the corresponding model. Geometries E–H show halobenzene interactions with imidazole, while geometries I–L show interactions with indole. Outliers of the phenol dataset: (A) Iodobenzene interaction with d_I···π‑plane_ = 2.32 Å and α_C–I···⊥(π‑plane)_ = 56.9°. The gray dashed line indicates a π···π-interaction. (B) Iodobenzene interaction with d_I···π‑plane_ = 2.28 Å and α_C–I···⊥(π‑plane)_ = 59.8°. The gray dashed line indicates a π···π-interaction. (C) Bromobenzene interaction with d_Br···π‑plane_ = 2.28 Å and α_C–Br···⊥(π‑plane)_ = 58.0°. The gray dashed line indicates a C–H···π contact. (D) Iodobenzene interaction with d_I···π‑plane_ = 2.20 Å and α_C–I···⊥(π‑plane)_ = 52.8°. The gray dashed line indicates a C–H···π contact. Outliers of the imidazole dataset: (E) Iodobenzene interaction with d_I···π‑plane_ = 2.26 Å and α_C–I···⊥(π‑plane)_ = 48.0°. The gray dashed line indicates a C–H···π contact. (F) Iodobenzene interaction with d_I···π‑plane_ = 2.38 Å and α_C–I···⊥(π‑plane)_ = 59.0°. The gray dashed line indicates a C–H···π contact. (G) Iodobenzene interaction with d_I···π‑plane_ = 2.20 Å and α_C–I···⊥(π‑plane)_ = 57.5°. The gray dashed line indicates a C–H···π contact. (H) Iodobenzene interaction with d_I···π‑plane_ = 2.21 Å and α_C–I···⊥(π‑plane)_ = 54.5°. The gray dashed line indicates a C–H···π contact. Outliers of the indole dataset: (I) Iodobenzene interaction with d_I···π‑plane_ = 2.30 Å and α_C–I···⊥(π‑plane)_ = 57.1°. The gray dashed line indicates a π···π interaction. (J) Iodobenzene interaction with d_I···π‑plane_ = 2.13 Å and α_C–I···⊥(π‑plane)_ = 56.5°. The gray dashed line indicates a π···π interaction. (K) Bromobenzene interaction with d_Br···π‑plane_ = 2.10 Å and α_C–Br···⊥(π‑plane)_ = 48.5°. The gray dashed line indicates a C–H···π contact. (L) Chlorobenzene interaction with d_Cl···π‑plane_ = 2.38 Å and α_C–Cl···⊥(π‑plane)_ = 52.9°. The gray dashed line indicates a C–H···π interaction.

**Table 1:** Overview of Energy Values and Geometric Parameters for Outlier Structures of the Random Geometry Set of Phenol (A–D), Imidazole (E–H), and Indole (I–L), Highlighted in Figure 1 and Depicted in Figure 2 ^a^

	halogen	π-system	Δ*E*_calc_ [kJ/mol]	ΔE_pred_ [kJ/mol]	ΔΔE [kJ/mol]	d_X···_π_–plane_ [Å]	α_(C‑X···_⊥(π_–plane))[_°, deg]
A	I	phenol	–25.83	–16.38	–9.45	2.32	56.88
B	I	phenol	–14.61	1.97	–16.58	2.28	59.85
C	Br	phenol	–7.38	–21.35	13.97	2.28	57.96
D	I	phenol	5.47	–21.63	27.11	2.20	52.76
E	I	imidazole	–22.83	–12.43	–10.40	2.26	48.00
F	I	imidazole	–20.53	4.49	–25.01	2.38	58.97
G	I	imidazole	–8.65	–17.24	8.59	2.20	57.51
H	I	imidazole	7.30	–15.83	23.13	2.21	54.55
I	I	indole	–24.76	–7.04	–17.72	2.30	57.57
J	I	indole	–12.55	8.18	–20.73	2.13	56.54
K	Br	indole	–10.70	–28.41	17.71	2.10	48.47
L	Cl	indole	2.94	–27.15	30.09	2.38	52.94

a The halogen symbolizes the interacting halobenzene. Values of calculated and predicted energies, as well as the difference between both, are given in kJ/mol. Distance values d_X···π‑plane_ between the halogen and the π-plane are given in Å. Angle values between the C-X vector and the normal to the π-plane are given in degrees [°, deg].

Example A corresponds to a complex formed between iodobenzene and phenol and exhibits a highly compact interaction geometry. The iodine atom is positioned at a distance of d_I···π‑plane_ = 2.32 Å and an angle of α_C–I···⊥(π‑plane)_ = 56.9°. For this configuration, the predicted adduct formation energy deviates strongly from the reference value, yielding an energy difference of ΔΔ*E*(A) = −9.45 kJ/mol (Δ*E*_calc_ = −25.83 kJ/mol, Δ*E*_pred_ = −16.38 kJ/mol), indicating a pronounced underestimation of the interaction strength by the model. This discrepancy can be attributed to the fact that the corresponding geometric descriptors lie well outside the region of the feature space sampled during training. This is reflected by a Mahalanobis distance that is more than three times higher than that of the distribution center. Comparison with the training set confirms this assumption because the iodine···π-plane separation in Example A is markedly shorter than the minimum distance encountered during training (*d*_min(X···π‑plane)_(training) = 2.75 Å), while the associated torsion angle substantially exceeds the largest value present in the training data (α_max(C‑X···⊥(π‑plane))_(training) = 40°). In addition, the proximity of the halogen atom to the phenol ring, and in particular to the hydroxyl substituent, may introduce stabilizing contributions not adequately captured by the model, potentially arising from interaction between the negatively charged region surrounding the halogen and the nearby hydrogen atom. The mutual arrangement of the aromatic rings may further stabilize the complex through a π···π interaction.

Example B exhibits a comparable trend, with the predicted interaction energy again markedly underestimated (ΔΔ*E*(B) = −16.58 kJ/mol). This discrepancy arises from a qualitative misclassification of the interaction, as the calculated and predicted energies differ not only in magnitude but also in sign (Δ*E*_calc_ = −14.61 kJ/mol, Δ*E*_pred_ = 1.97 kJ/mol), indicating that the stabilizing π···π contribution is not adequately represented by the model. As in Example A, the associated distance and angle values fall well outside the range sampled during training, indicating a closely related but distinct geometric representation. In this configuration, the mutual alignment of the aromatic rings enhances the π···π interaction character even further. The resulting prediction error highlights a broader limitation of the model, which appears insufficiently able to generalize such highly compact, strongly tilted geometries that enable favorable π···π or X···H interaction motifs, thereby leading to a systematic underestimation of their stabilizing energetic contributions.

Examples C and D show the opposite trend compared to A and B, with the interaction energies strongly overestimated by the model. In Example C, the calculated energy is moderately stabilizing (Δ*E*_calc_ = −7.38 kJ/mol), whereas the model predicts a substantially stronger interaction (Δ*E*_pred_ = −21.35 kJ/mol), resulting in a large positive deviation (ΔΔ*E*(C) = 13.97 kJ/mol). An even more pronounced discrepancy is observed for Example D, where the calculated energy indicates a slightly repulsive interaction (Δ*E*_calc_ = 5.47 kJ/mol), while the model predicts a strongly attractive complex (Δ*E*_pred_ = −21.63 kJ/mol).

In both geometries, the halogen approaches the phenol π-system at very short distances and with pronounced tilt angles, placing these structures outside the range of configurations represented in the training data. While such compact arrangements may still resemble stabilizing contacts, the reference calculations indicate that short-range repulsive contributions can become significant in these cases. The inability of the model to capture this repulsive part suggests that it primarily extrapolates attractive interactions from short contact distances, thereby failing to account for steric and electronic repulsion at close approach.

In the present case, all four examples fall into category (a), exhibiting features not covered during training and correspondingly high MD values. Because these atypical and often repulsive interaction geometries were not included in the training set, the model lacks the necessary information to penalize them appropriately. The same applies to the recognition of π···π interactions and their beneficial contribution. While accurately modeling these configurations is not essential, since they are typically identified and filtered out earlier or handled by dedicated repulsion terms, future models may benefit from including representative repulsive σ-hole or π···π arrangements to further enhance robustness and transferability.

### Imidazole Model Evaluation

The imidazole model was evaluated using the same two-step procedure. For the constrained subset (step (i), N = 16,075), the model achieved high accuracy (R^2^ = 0.9848, RMSE = 0.34 kJ/mol) with ΔΔ*E* between +3.71 kJ/mol and −3.17 kJ/mol (9.42% beyond ±0.5 kJ/mol). When applied to the full test set (step (ii), N = 57,297), the performance remained strong with R^2^ = 0.9096 and RMSE = 0.94 kJ/mol, although the spread of deviations widened, with ΔΔ*E* values ranging from +25.01 kJ/mol to −24.29 kJ/mol (19.28% beyond ±0.5 kJ/mol).

A corresponding parity plot of the imidazole results is shown in Figure 1. Similar to the phenol model, most data points cluster along the parity line, while a small number of higher-MD structures exhibit larger prediction errors. Four extreme outliers (labeled E–H) are examined in detail (Figure 2). Calculated and predicted adduct formation energies, energy differences, distances d_X···π‑plane_ between the halogen and the π-plane, as well as the angle between the C-X vector and the normal to the π-plane α_C‑X···⊥(π‑plane)_ of these imidazole outliers, are listed in Table 1.

Examples E–H exhibit closely related interaction geometries characterized by strongly tilted halobenzene orientations and halogen positions located near the boundaries of the model’s training grid. In all four cases, the halogen atom resides outside the π-plane, and the dominant stabilizing motif is limited to C–H···π contacts rather than direct halogen···π interactions. The primary geometric differences among these structures arise from the specific positioning of the halogen, which, in turn, governs the relative placement of the C–H···π and H···X contacts. Despite their overall similarity, three of the four geometries (E–G) correspond to attractive interactions, whereas example H is predicted to be repulsive by the reference calculation. A particularly instructive comparison can be made between examples E and H, which share nearly identical orientations but differ subtly in distance and angular values. In example E, the slightly larger contact distance and smaller interaction angle give rise to a strongly stabilizing interaction, while the modest geometric changes observed in example H shift the balance toward repulsion with a shorter distance and larger angle. This sharp transition from attractive to repulsive behavior occurs over a narrow region of geometric space that is sparsely sampled in the training data and is therefore not reliably captured by the model. It should be emphasized that the geometries discussed here represent only a limited subset, selected from the “worst-case” identified outliers, and are not intended to be exhaustive. They serve as illustrative examples highlighting characteristic failure sources of the model rather than a comprehensive description of all deviations present in the test set. In particular, although not shown explicitly, the imidazole-derived data also contain outlier geometries involving alternative interaction motifs, such as π···π contacts, which may give rise to similar prediction challenges. In summary, the imidazole model demonstrates performance analogous to the phenol model, with closely related error characteristics and limitations, but retains good overall agreement between calculated and predicted energies.

### Indole Model Evaluation

The indole model showed similarly strong performance under the two-step evaluation framework. Within the constrained region (step (i), N = 15,211), the model achieved excellent agreement with reference data (R^2^ = 0.9953, RMSE = 0.28 kJ/mol) and notably low energy deviations between +2.72 kJ/mol and −2.36 kJ/mol (6.94% beyond ±0.5 kJ/mol). Evaluation on the unconstrained full dataset (step (ii), N = 55,415) resulted in a decrease to R^2^ = 0.9269 and RMSE = 1.17 kJ/mol, with deviations of ΔΔ*E* between +34.47 kJ/mol and −20.73 kJ/mol (23.36% beyond ±0.5 kJ/mol). This indicates that the indole feature space contains a broader distribution of challenging geometries when the distance and angle constraints are removed and could be due to the larger size of the acceptor system.

The corresponding parity plot of the indole results (Figure 1) again highlights four outliers (I–L), which exhibit the largest discrepancies. All individual values of these four indole outliers can be found in Table 1. As with the phenol and imidazole models, these structures correspond to geometries lying well outside the training distribution, consistent with elevated MD values.

Examples I–L illustrate characteristic limitations of the indole model that are closely related to those observed for phenol and imidazole. Examples I and J feature pronounced π···π interaction motifs and correspond to strongly stabilizing reference energies that are not adequately captured by the model. Although both geometries exhibit similarly large tilt angles, small variations in the intermolecular distance lead to qualitatively different predictions. While example I, with a slightly larger separation, is still predicted to be attractive, example J, characterized by a shorter contact distance, is incorrectly classified as repulsive. This behavior indicates limited generalizability of the model with respect to distance variations in compact π···π arrangements and distances beyond the feature space during training.

In contrast, examples K and L are dominated by C–H···π interactions at short contact distances and high angular values. In these cases, the balance between stabilizing H···X interactions and short-range repulsive contributions is particularly delicate. A narrow geometric transition separates attractive configurations, where favorable H···X contacts prevail, from repulsive arrangements dominated by steric C–H clashes. This subtle crossover occurs over a region of feature space that is insufficiently represented in the training data and, is therefore, not reliably reproduced by the model.

Thus, the general trend across all three models is that prediction quality decreases as the geometries diverge further from the region sampled during training, particularly in repulsive or otherwise atypical configurations such as π···π or C–H···π arrangements.

### PDB Scan for Halogen···π Interactions in Crystal Structures

In a previous study, we performed a comprehensive PDB scan to identify halogen···π interactions involving phenylalanine.^48^ Building on that work, we expanded the analysis to focus on the aromatic side chains of tyrosine, histidine, and tryptophan. 239,149 crystal structures (as of July 2025) were analyzed, with 9810 unique PDB IDs containing chlorine-, bromine-, or iodine-bearing ligands. Phenylalanine accounts for over 40% of all observed contacts (already analyzed in our earlier work),^48^ followed by tyrosine at 29.36%. Histidine (15%) and tryptophan (12%) are less frequently encountered but still represent relevant biological environments for σ-hole interactions. This result is not unexpected, as Trp is encoded only by one DNA triplet in comparison to two DNA triplets for Phe, Tyr, and His. Recent analyses of amino acid frequencies in human PDB structures and human proteins in the UniProt database highlight that Trp is about half as frequent as His, while Phe and Tyr are slightly more frequent than His.^51^ While the trends are quite similar in our results, it is evident that His is less frequently found in close proximity to halogens compared to its statistical occurrence in human proteins of the pdb. However, this is most likely due to the multitude of other interactions His can exhibit, such as hydrogen bonding, charged interactions, or metal coordination. Results of the scan and a more detailed analysis of the individual amino acid values are shown in Table 2.

**Table 2:** PDB Scan Results for Halogen···π Contacts and Halogen···π Interactions^a^

Addressed AA	Halogen	Halogen···π contacts within 5 A (% of total)	Halogen···π interactions after applied filters (% of contacts)
Histidine	Cl, Br, I	3597 (15.28%)	661 (18.38%)
Phenylalanine	Cl, Br, I	10174 (43.23%)	1114 (10.95%)
Tryptophan	Cl, Br, I	2855 (12.13%)	384 (13.45%)
Tyrosine	Cl, Br, I	6910 (29.36%)	1152 (16.67%)
Total		23536	3311 (14.07%)
Tyrosine	Cl	5275 (76.34%)	856 (74.30%)
Tyrosine	Br	1232 (17.83%)	225 (19.54%)
Tyrosine	I	403 (5.83%)	71 (6.16%)
Histidine	Cl	2966 (82.46%)	602 (91.07%)
Histidine	Br	489 (13.59%)	50 (7.56%)
Histidine	I	142 (3.95%)	9 (1.37%)
Tryptophan	Cl	2150 (75.31%)	296 (77.08%)
Tryptophan	Br	567 (19.86%)	76 (19.79%)
Tryptophan	I	138 (4.83%)	12 (3.13%)

a Results of Interactions with Tyrosine, Histidine, and Tryptophan are Reported for Each Halogen Separately.

To isolate interactions characteristic of the halogen’s σ-hole engagement, we applied distance and angular criteria and additionally required the C-X bond vector of the ligand to point toward the π-plane, an orientation consistent with the formation of a directional σ-hole contact. After applying these geometric filters, we identified N_TYR_ = 1152, N_HIS_ = 661, and N_TRP_ = 384 halogen···π interactions for further analysis.

For consistent comparison across systems, a matched-molecular-pair approach was employed. Each ligand halogen donor was substituted with the corresponding halobenzene model system. The halogen atom and C-X vector were aligned exactly with the original ligand geometry, and the benzene ring was superimposed onto the ligand’s aromatic ring to preserve orientation. Likewise, the amino acid side chain was truncated at the C_γ_–C_β_ bond and replaced with the protonated, optimized model system by aromatic-ring superposition. This substitution strategy necessarily neglects ligand-specific electronic tuning of halogen strength but ensures consistent geometric comparison. MP2/TZVPP single-point interaction energies were computed using TURBOMOLE. Repulsive geometries with interaction energies exceeding +10 kJ/mol were discarded, resulting in final datasets of N_TYR_ = 1142, N_HIS_ = 660, and N_TRP_ = 384 geometries. Although the datasets describe halobenzene-phenol, -imidazole, and -indole interactions, we refer to them as the “tyrosine,” “histidine,” and “tryptophan” datasets, respectively, to distinguish the PDB-derived sets from the randomly generated ones.

### Model Evaluation on PDB-Derived Sets

#### Addressing the Side Chain of Tyrosine

For the 1142 PDB-derived halogen···TYR geometries (denoted as “tyrosine set”), features were extracted and submitted to the phenol-based NN model. In the restricted evaluation (step (i) 578 structures lie within the training distribution (2.75 Å ≤ d_X···π‑plane_ ≤ 4.5 Å, α_C‑X···⊥(π‑plane)_ ≤ 40°). Within this domain, the model achieved excellent accuracy (R^2^_TYR(i)_ = 0.9904, RMSE_TYR(i)_ = 0.30 kJ/mol), with deviations confined to +0.71 to −1.58 kJ/mol, with 8.13% beyond ±0.5 kJ/mol (Figure S4). These results confirm that the model interpolates reliably and is not dependent on memorization. In the full-data evaluation (step (ii) model performance decreased slightly but remained strong (R^2^_TYR(ii)_ = 0.9530, RMSE_TYR(ii)_ = 0.76 kJ/mol). Although a few interactions exhibit larger discrepancies (up to +8.33 and −3.89 kJ/mol), these values show a steep improvement, where the positive deviations drop rapidly from +8.33 kJ/mol to +3 kJ/mol. Similarly, the negative tail of differences contracts below −2 kJ/mol after a few examples. Still, 20.23% lie beyond ±0.5 kJ/mol, but most structures remain accurately predicted (Figure 3, Tyrosine Set).

**Figure 3:**
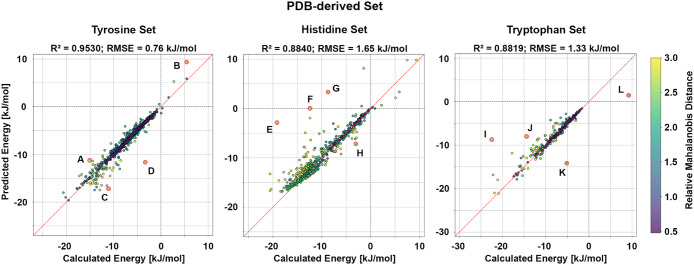
Model performance on the PDB-derived set. Calculated adduct formation energies are plotted against the corresponding predicted values for the phenol, imidazole, and indole models. The phenol model achieves an R^2^_TYR_ = 0.9530 with an RMSE_TYR_ = 0.76 kJ/mol, the imidazole model achieves an R^2^_HIS_ = 0.8840 with an RMSE_HIS_ = 1.65 kJ/mol, and the indole model achieves an R^2^_TRP_ = 0.8819 with an RMSE_TRP_ = 1.33 kJ/mol. The red dashed line denotes perfect agreement between calculated and predicted energies, while the gray dashed lines mark the transition between negative and positive energies. Data points are colored according to their relative MD, as indicated by the color scale. Data points highlighted by red circles and labeled A–L correspond to selected outliers and are discussed in detail in Figure 4.

Representative outliers (Figure 4, examples A–D) exhibit large Mahalanobis distances, indicating that their geometric features lie at or beyond the boundaries of the training distribution, as previously described. Calculated and predicted adduct formation energies, energy differences, distances d_X···π‑plane_ between the halogen and the π-plane, as well as the angle between the C-X vector and the normal to the π-plane α_C‑X···⊥(π‑plane)_ of all subsequent outliers are listed in Table 3. Examples A and B are dominated by π···π-type arrangements characterized by strongly tilted halobenzene orientations and very short halogen···π-plane separations. In example A, the exceptionally small distance of 1.10 Å places the halogen in close proximity to the aromatic system, enabling not only a favorable π···π interaction but also an additional stabilizing side interaction involving the halogen. Despite this, the interaction energy is underestimated by the model. In contrast, example B exhibits a similar tilt angle but a larger distance of 1.54 Å. Here, the halogen is positioned such that its negatively charged belt approaches the phenolic oxygen atom, leading to repulsive contributions that reduce the overall stabilization. It should be noted that the orientation of the hydroxyl group in these PDB-derived structures may differ from that in the optimized phenol reference configuration. A rigorous treatment of such effects would require more extensive and computationally demanding preprocessing.

**Figure 4:**
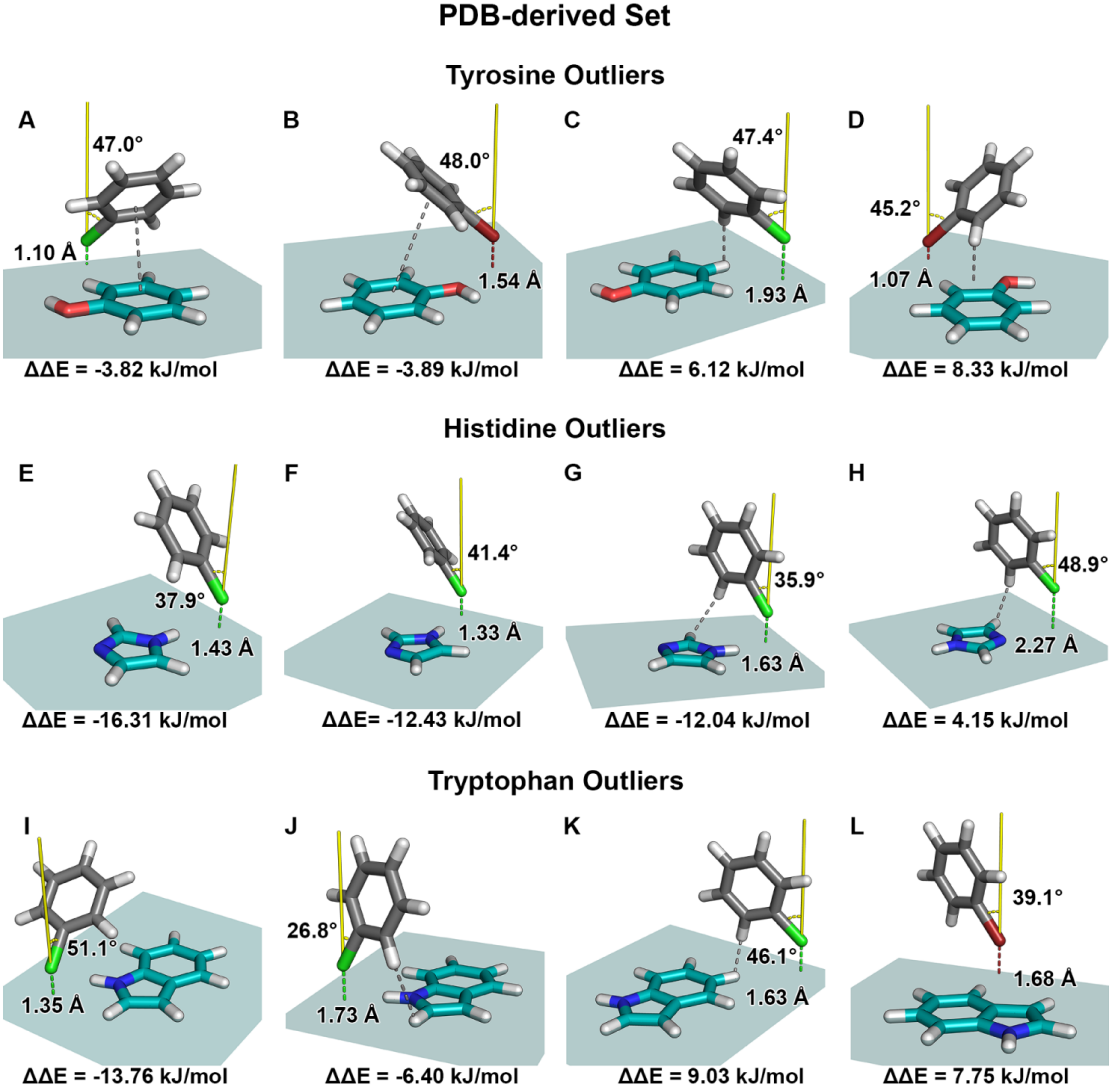
Interaction geometries for selected outliers from the PDB-derived geometry set involving the phenol, imidazole, and indole side chains of tyrosine, histidine, and tryptophan, as identified in the scatter plot shown in Figure 3. These structures correspond to data points exhibiting large deviations between the calculated and predicted adduct formation energies. Geometries A–D depict the relative orientation of the halobenzene (gray) with respect to the phenol π-system (teal). Shown are the halogen distance, d_X···π‑plane_ (dashed line, colored according to the halogen), to the π-system plane in Å, the torsion angle, α_C‑X···⊥(π‑plane)_ (yellow), between the C-X bond vector and the normal to the π-system plane in degrees, and the corresponding energy difference, ΔΔ*E*. The gray dashed line indicates the type of interaction that may contribute to the observed prediction error. For clarity, the teal plane also illustrates the extent of the training grid used in the corresponding model. Geometries E–H show halobenzene interactions with imidazole, while geometries I–L show interactions with indole. Outliers of the tyrosine dataset: (A) Chlorobenzene interaction with d_Cl···π‑plane_ = 1.10 Å and α_C–Cl···⊥(π‑plane)_ = 47.0°. The gray dashed line indicates a π···π-interaction. (B) Bromobenzene interaction with d_Br···π‑plane_ = 1.54 Å and α_C–Br···⊥(π‑plane)_ = 48.0°. The gray dashed line indicates a π···π-interaction. (C) Chlorobenzene interaction with d_Cl···π‑plane_ = 1.93 Å and α_C–Cl···⊥(π‑plane)_ = 47.4°. The gray dashed line indicates a C–H···π contact. (D) Bromobenzene interaction with d_Br···π‑plane_ = 1.07 Å and α_C–Br···⊥(π‑plane)_ = 45.2°. The gray dashed line indicates a C–H···π contact. Outliers of the histidine dataset: (E) Chlorobenzene interaction with d_Cl···π‑plane_ = 1.43 Å and α_C–Cl···⊥(π‑plane)_ = 37.9°. (F) Chlorobenzene interaction with d_Cl···π‑plane_ = 1.33 Å and α_C–Cl···⊥(π‑plane)_ = 41.4°. (G) Chlorobenzene interaction with d_Cl···π‑plane_ = 1.63 Å and α_C–Cl···⊥(π‑plane)_ = 35.9°. The gray dashed line indicates a C–H···π contact. (H) Chlorobenzene interaction with d_Cl···π‑plane_ = 2.27 Å and α_C–Cl···⊥(π‑plane)_ = 48.9°. The gray dashed line indicates a C–H···π contact. Outliers of the tryptophan dataset: (I) Chlorobenzene interaction with d_Cl···π‑plane_ = 1.35 Å and α_C–Cl···⊥(π‑plane)_ = 51.1°. (J) Chlorobenzene interaction with d_Cl···π‑plane_ = 1.73 Å and α_C–Cl···⊥(π‑plane)_ = 26.8°. The gray dashed line indicates a π···π interaction. (K) Chlorobenzene interaction with d_Cl···π‑plane_ = 1.63 Å and α_C–Cl···⊥(π‑plane)_ = 46.1°. The gray dashed line indicates a C–H···π contact. (L) Bromobenzene interaction with d_Br···π‑plane_ = 1.68 Å and α_C–Br···⊥(π‑plane)_ = 39.1°.

**Table 3:** Overview of Energy Values and Geometric Parameters for Outlier Structures of the PDB-Derived Set of Tyrosine (Phenol, A–D), Histidine (Imidazole, E–H), and Tryptophan (Indole, I–L), Highlighted in Figure 3 and Depicted in Figure 4 ^a^

	halogen	addressed AA	PDB ID	Δ*E*_calc_ [kJ/mol]	ΔE_pred_ [kJ/mol]	ΔΔE [kJ/mol]	d_X···_π_–plane_ [Å]	α_(C‑X···_⊥(π_–plane))_ [°, deg]
A	Cl	TYR	6TOM	–15.08	–11.26	–3.82	1.1	46.97
B	Br	TYR	6FY4	5.45	9.34	–3.89	1.54	48.02
C	Cl	TYR	3UIC	–11.08	–17.19	6.12	1.93	47.45
D	Br	TYR	2WP5	–3.33	–11.66	8.33	1.07	45.17
E	Cl	HIS	3VAD	–19.16	–2.86	–16.31	1.43	37.92
F	Cl	HIS	9BST	–12.40	0.03	–12.43	1.33	41.36
G	Cl	HIS	2VPY	–8.70	3.34	–12.04	1.63	35.95
H	CL	HIS	6BYA	–3.02	–7.17	4.15	2.27	48.86
I	Cl	TRP	5LIK	–22.50	–8.74	–13.76	1.35	51.06
J	Cl	TRP	5IF2	–14.42	–8.02	–6.40	1.73	26.77
K	Cl	TRP	3DQT	–5.13	–14.16	9.03	1.63	46.09
L	Br	TRP	9EWA	9.19	1.44	7.75	1.68	39.13

a The halogen symbolizes the interacting halobenzene. The PDB ID indicates the crystal structure from which the interaction geometry was extracted. Values of calculated and predicted energies, as well as the difference between both, are given in kJ/mol. Distance values d_X···π‑plane_ between the halogen and the π-plane are given in Å. Angle values between the C-X vector and the normal to the π-plane are given in degrees [°, deg].

Examples C and D are instead governed primarily by C–H···π motifs. In example C, the geometry represents an intermediate case between a C–H···π contact and a π···π-like arrangement. Although the C–H positioning alone would suggest increased repulsion due to short H···H contact, the combined interaction pattern remains stabilizing overall, as reflected by the calculated interaction energy of Δ*E*_calc_ = −11.08 kJ/mol. In example D, both a side-on H···X interaction and a close approach of a halobenzene hydrogen toward the hydroxyl oxygen are observed. However, the overall geometry is excessively compact, leading to increased short-range repulsion and only weak net stabilization in the reference calculation (Δ*E*_calc_ = −3.33 kJ/mol). In contrast, the model predicts a much stronger attraction, indicating that repulsive contributions in such crowded geometries are not adequately captured.

Crucially, these interaction motifs fall outside the structural patterns characteristic of σ-hole contacts. The halogen frequently approaches from an off-axis position relative to the π-plane, or the halobenzene ring is positioned at unrealistically short distances, inducing either H···X contacts or steric crowding and positive Δ*E*_calc_ values. Furthermore, the C–X bond is typically oriented away from the aromatic system, making the directional geometry required for σ-hole interactions unattainable.

#### Addressing the Side Chain of Histidine

The PDB-derived halogen···HIS dataset contained 660 interactions. Of these, 128 structures satisfied the training-space restrictions (step (i)). Within this subset (denoted as “histidine set”), the imidazole model performed very well, achieving R^2^_HIS(i)_ = 0.9834 and RMSE_HIS(i)_ = 0.43 kJ/mol, with deviations between +1.46 kJ/mol and −1.36 kJ/mol (21.09% beyond ±0.5 kJ/mol). Evaluation on the full His dataset (step (ii)) produced a moderate decline in accuracy (R^2^_HIS(ii)_ = 0.8840, RMSE_HIS(ii)_ = 1.65 kJ/mol, 51.44% beyond ±0.5 kJ/mol; Figure S4). Outliers reached +4.15 kJ/mol and −16.31 kJ/mol, but the distribution shows similar behavior to the tyrosine case: positive deviations drop sharply after a few large positive values (to <2.5 kJ/mol), and only 18 structures exhibit negative deviations of more than −4 kJ/mol (Figure 3, Histidine Set).

Outlier analysis (Figure 4, examples E and H) confirms that these cases correspond to geometries far from the feature patterns represented during training, as reflected by high MDs. Individual energy, distance, and angle values are listed in Table 3. Many outliers display distorted orientations of the C-X bond relative to the imidazole π-system and comparatively short halogen···π-plane separations. Across this series, the reference interaction energies become progressively less favorable from E to H. For examples E–G, the interaction energies are consistently underestimated by the model, whereas example H exhibits the opposite behavior and is overestimated. Notably, these geometries display smaller angle values than those observed for the phenol-derived outliers, yet substantial prediction errors persist. This indicates that the very short intermolecular distances alone are sufficient to place these structures outside the region of feature space sampled during training. Consequently, even when only one of the two key geometric descriptors—distance or angle—deviates strongly from the training distribution, the model struggles to reliably capture the balance between stabilizing and repulsive contributions. These results highlight a sensitivity of the imidazole model to compact C–H···π arrangements that are insufficiently represented in the training data.

#### Addressing the Side Chain of Tryptophan

384 halogen···TRP interactions were identified (denoted as the ″tryptophan set”), of which 96 fell within the training distribution (step (i)). In this region, the indole model achieved excellent accuracy (R^2^_TRP(i)_ = 0.990, RMSE_TRP(i)_ = 0.33 kJ/mol) with differences ranging between +1.18 kJ/mol and −0.38 kJ/mol (13.54% beyond ±0.5 kJ/mol). On the full dataset (step (ii)), performance decreased to R^2^_TRP(ii)_ = 0.8819 and RMSE_TRP(ii)_ = 1.33 kJ/mol, with the largest deviations reaching +9.03 kJ/mol and −13.76 kJ/mol (28.65% beyond ±0.5 kJ/mol; Figure S4). As before, these values represent a small minority: positive deviations fall rapidly after the two largest examples to <2 kJ/mol, and only a few structures contribute to the most negative tail >−6 kJ/mol (Figure 3, Tryptophan Set).

The outliers (Figure 4, examples I–L) again correspond to geometries with elevated MDs, reflecting substantial deviation from the training distribution. These structures typically feature improper interaction angles, nonaxial halogen orientations, or sterically implausible proximities to the indole ring, thus displaying configurations inconsistent with physically meaningful σ-hole interactions. Examples I and J are characterized by underestimated interaction energies, with example I exhibiting a pronounced π···π interaction motif, while example J is dominated by a C–H···π contact. In the latter case, short H···H contacts would suggest an increased repulsive contribution. However, this effect is likely at least partially compensated for by a favorable H···X interaction, resulting in a net attractive reference energy. In contrast, examples K and L show overestimated interaction energies and are governed by weak C–H···π contacts or, in the case of example L, by the absence of a clearly identifiable stabilizing interaction motif.

Despite these differences, all four structures share very short halogen···π-plane distances, while the corresponding interaction angles span a wide range.

### Enhancing the Performance by Additional Training Data

Outlier analysis across all test sets and all three models indicates that poor predictive performance is consistently associated with elevated MD values. This relationship suggests that these poorly predicted interaction geometries possess features located at the boundaries of, or outside, the original training set distribution. These observations are consistent with our previous findings.^48^ A commonly recognized advantage of neural network models is their modular expandability, both in terms of the learning architecture and the training data that can be incorporated during model development.

To evaluate this property in the present context, we retrained each model using an augmented training set that included an additional 300,000 randomly generated interaction geometries (100,000 per halogen, a similar procedure was used for the previous random geometry test sets). The underlying assumption is that broadening the training distribution enables the models to capture a wider range of structural features, thereby improving their ability to generalize to previously unseen or sparsely represented regions of feature space. A broader distribution should enhance the models’ capacity to interpolate between data points and, consequently, reduce the error associated with examples that were previously identified as outliers.

All model configurations were kept unchanged, and each of the three systems—phenol, imidazole, and indole—was retrained using the expanded dataset. The resulting training performance remained high, with the retrained models yielding R^2^ values of 0.9958, 0.9827, and 0.9952, and RMSE values of 0.34, 0.45, and 0.39 kJ/mol for phenol, imidazole, and indole, respectively. These values are slightly lower than those in the original training runs, which may be attributed to the broadened feature distribution: the newly introduced random geometries are inherently less systematically sampled and therefore comparatively underrepresented relative to the structured data present in the initial training set. As a result, the optimization process converges toward parameter sets that best describe the combined distribution on average, moderately reducing the apparent fit quality of the training data. Nevertheless, the central objective of this retraining step is to enhance the models’ capacity for generalization, particularly for configurations previously identified as difficult to predict.

To assess this effect, the retrained models were first evaluated on the random geometry test set, exactly as used before. The updated performance metrics indicate improved predictive quality across all systems, with new R^2^ values of 0.9851, 0.9555, and 0.9803, and RMSE values of 0.58, 0.86, and 0.80 kJ/mol for phenol, imidazole, and indole, respectively (Figure 5). These results demonstrate a pronounced reduction in prediction error for interaction geometries located near the boundaries of the original feature space, supporting the conclusion that the broadened training distribution enables more reliable interpolation in regions that were previously sparsely sampled. To illustrate the resulting improvement in predictive performance, the outlier geometries discussed above are highlighted in Figure 5. In contrast to the original models, these data points now cluster closely around the parity line, indicating a substantial enhancement in agreement between calculated and predicted energies.

**Figure 5:**
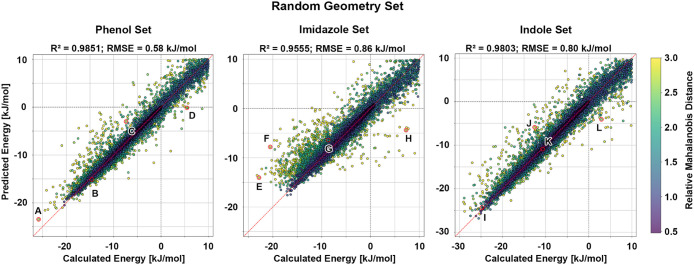
Model performance on the random geometry set after each model: Calculated adduct formation energies are plotted against the corresponding predicted values for the retrained phenol, imidazole, and indole models. The phenol model achieves an R^2^ = 0.9851 with an RMSE = 0.58 kJ/mol, the imidazole model achieves an R^2^ = 0.9555 with an RMSE = 0.86 kJ/mol, and the indole model achieves an R^2^ = 0.9803 with an RMSE = 0.80 kJ/mol. The red dashed line denotes perfect agreement between calculated and predicted energies, while the gray dashed lines mark the transition between negative and positive energies. Data points are colored according to their relative MD, as indicated by the color scale. Data points highlighted by red circles and labeled A–L correspond to the previously discussed selected outliers (Figures 1 and 2).

To further validate the generalization behavior, the retrained models were subsequently reevaluated on the PDB-derived test set. Again, performance improvements were observed, with phenol, imidazole, and indole models achieving new R^2^ values of 0.9610, 0.9252, and 0.9179, and corresponding RMSE values of 0.69, 1.35, and 1.11 kJ/mol. A complete overview of all R^2^ and RMSE values of all models and evaluation steps of the different datasets is summarized in Table S3. Comparison with earlier results shows that the predicted energies now align more closely with the calculated reference values, with data points shifting noticeably toward the parity line in all three models (Figure 6). However, it is also evident that a subset of data points deviates further from the parity line than before. This behavior is consistent with the broader training distribution introduced during retraining. Because the model now optimizes its parameters to achieve the best average performance across a more diverse set of geometries, certain regions of the original systematic dataset become slightly less favored in the fitting process. These data points, therefore, incur a modest loss in accuracy as a consequence of the model’s increased generalization. In principle, this effect could be mitigated by more carefully designing or weighting the additional training geometries, but such optimization and further refinement lie beyond the scope of the present study.

**Figure 6:**
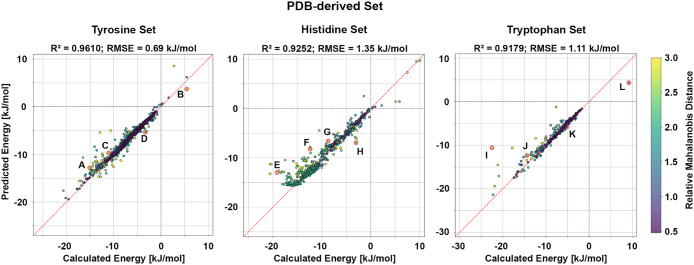
Model performance on the PDB-derived set after each model: Calculated adduct formation energies are plotted against the corresponding predicted values for the retrained phenol, imidazole, and indole models. The phenol model achieves an R^2^_TYR_ = 0.9610 with an RMSE_TYR_ = 0.69 kJ/mol, the imidazole model achieves an R^2^_HIS_ = 0.9252 with an RMSE_HIS_ = 1.35 kJ/mol, and the indole model achieves an R^2^_TRP_ = 0.9179 with an RMSE_TRP_ = 1.11 kJ/mol. The red dashed line denotes perfect agreement between calculated and predicted energies, while the gray dashed lines mark the transition between negative and positive energies. Data points are colored according to their relative MD, as indicated by the color scale. Data points highlighted by red circles and labeled A–L correspond to the previously discussed selected outliers (Figures 3 and 4).

Taken together these findings indicate that expanding the training set with randomly generated interaction geometries not only increases model robustness but also significantly enhances the predictive accuracy for previously challenging regions of the interaction space.

## Conclusion and Outlook

In the present work, we significantly extend our previous proof-of-concept study on neural network-based prediction of halogen···π interaction energies by moving from a single aromatic model system to a family of biologically relevant aromatic amino acid side chains. Dedicated models were developed for phenol, imidazole, and indole as representative models of tyrosine, histidine, and tryptophan, respectively. For each system, large, systematically generated training sets were constructed, and high-level MP2/TZVPP reference energies were used to train feed-forward neural networks on compact, rotationally and translationally invariant geometric descriptors. Across all three models, excellent predictive performance was achieved within the training domain, with R^2^ values consistently above 0.98 and RMSE values well below 0.5 kJ/mol, demonstrating that the chosen feature representations and model architectures are well-suited to capture directional halogen···π interactions. Learning curve analysis further indicates that model performance converges rapidly with training set size, with near-optimal accuracy already achieved using roughly 30–40% of the available training data, while further data contribute to improved robustness.

A rigorous two-step evaluation strategy was employed to assess the generalization behavior both within and beyond the sampled feature space. While predictive accuracy remains high for geometries interpolating within the training distribution, systematic deviations were observed for compact or highly tilted configurations that lie outside the original training domain. Detailed outlier analysis revealed that these failures predominantly arise from interaction motifs not explicitly targeted during training, such as π···π or C–H···π contacts, or from short-range repulsive contributions that become dominant at very small intermolecular distances. Importantly, these limitations are consistent across all three aromatic systems and mirror trends already observed in our earlier phenylalanine-based model, underscoring the generality of the underlying behavior rather than model-specific artifacts.

Application of the models to PDB-derived interaction geometries further demonstrates their practical relevance. For geometries consistent with σ-hole-driven halogen···π contacts, the models reproduce reference interaction energies with high accuracy, confirming their suitability for analyzing experimentally observed protein–ligand complexes. Deviations observed for certain PDB-derived structures can largely be traced back to geometries that violate the directional or distance requirements of genuine σ-hole interactions or to sterically crowded arrangements where repulsive effects dominate. These findings highlight the importance of combining geometric prefiltering with machine-learning-based energy evaluation when applying such models in real-world structural biology contexts.

A key advantage of this framework is its modular expandability. By augmenting the training data with additional randomly generated geometries, we demonstrated that model robustness and generalization can be substantially improved without alteration of the underlying architecture. The retrained models exhibit a distinct enhanced performance for previously challenging regions of feature space, including both random test geometries and PDB-derived geometries, albeit at the cost of a minor reduction in peak accuracy within the original, densely sampled domain. This trade-off illustrates a central design consideration for machine-learning approaches: balancing local precision with global transferability. As the halogen bond-specific model published recently by Devore and Shuford.^52^ features interactions with ammonia as a standard acceptor, it unfortunately does not provide a valid comparison for our specialized model on halogen···π interaction energies. Thus, we turned to the more general-purpose machine-learned model (AIMNet2 ^53^), which was developed by Isayev and coworkers as a model that can, among many other applications, also recognize molecular interactions such as σ-hole interactions in halogen bonding. First results indicate that our highly specialized model is more suited for this particular application but is certainly limited to this specific task.

Taken together, this study establishes and confirms a scalable and transferable strategy for predicting halogen···π interaction energies across multiple aromatic environments with near-quantum-mechanical accuracy at negligible computational cost. By exploiting the “double jump” strategy from CCSD­(T) to MP2 and ultimately to neural networks across several chemically distinct models, this work moves a decisive step closer toward practical deployment in structure-based drug design. Because the models are trained on gas-phase interaction energies, they are not intended to directly predict binding free energies. Instead, they are designed to complement existing docking and scoring frameworks, where solvation, desolvation penalties, and entropic effects are treated through implicit solvent models and empirical scoring terms. Future developments will focus on (i) incorporating representative repulsive interaction motifs into the training process to improve behavior at short range, (ii) extending the framework to positively charged π-systems such as protonated histidine (imidazolinium) and arginine (guanidinium), which represent a distinct class of halogen···π interaction motifs, (iii) extending the approach to additional noncovalent interaction types, and (iv) integrating the resulting models into molecular docking and scoring frameworks such as PLANTS, as well as web-based analysis platforms. Ultimately, this class of physically informed, residue-specific machine-learning models offers a promising route toward a more accurate and interpretable treatment of halogen bonding effects in biomolecular recognition.

## Materials and Methods

### Structure Optimization

Geometry optimizations of the individual ligand model systems (iodobenzene, bromobenzene, and chlorobenzene) and the amino acid model systems (phenol, imidazole, and indole) were done at the MP2 ^54,55^ -level of theory using TURBOMOLE 7.7.1 ^56^ with a triple-ζ basis set (def2-TZVPP^57^) on the JUSTUS2 - bwHPC Cluster.^58^ Calculations were done in combination with the resolution of identity (RI) technique and the frozen core approximation. Frozen core orbitals were defined using default settings, where orbitals with energies below −3.0 au are considered core orbitals. The SCF convergence criterion was increased to 10^–8^ hartree. Relativistic effects for iodine were considered by an effective core potential (ECP).^59–67^

### Generation of Interaction Geometries

Interaction geometries of chloro-, bromo-, and iodobenzene in complex with phenol, imidazole, and indole were generated. To illustrate and describe the parameters of the geometry generation process, we subsequently used the complex of iodobenzene and phenol. Similar grids were generated for imidazole and indole interaction geometries with slightly adapted dimensions. The corresponding visualizations can be found in the Supporting Information (Figures S5 and S6). Halobenzenes were placed on a regular grid using X- and Z-translations (Figure 7a) for eight different distances (Figure 7b). Following previous approaches, an optimal σ-hole angle of α_C‑X···π‑plane_ = 180° was initially used. To capture rotational features, halobenzene itself was rotated around the *y*-axis. Furthermore, the σ-hole angle was altered to deviations from −40° to 40° in steps of 10° in the *x*- and *z*-directions (Figure 7d). Two additional rotation axes were incorporated, lying at 45° to the *x*- or *z*-axis (Figure 7e). Additionally, interaction geometries addressing phenol’s oxygen atom were generated in a spherical shape around the oxygen, where the C-X vector of the halobenzene points toward the oxygen atom with an angle of α_C‑X···O_ = 180° (Figure 7f). To capture different positions of the hydrogen atom attached to the oxygen, phenol’s O–H vector was altered in steps of 30° (Figure 7g). For imidazole complexes, additional interaction geometries addressing the nonprotonated nitrogen atom were generated in a spherical shape around the nitrogen, where the C-X vector of the halobenzene points toward the nitrogen atom with an angle of α_C‑X···N_ = 180°. Single-point calculations of more than 18.6 million (total sum of the three systems) interaction geometries were conducted at the MP2/TZVPP level of theory. Adduct formation energies were calculated as:

1 $$\Delta E=\left(E_{\mathrm{complex}}-\left(E_{\mathrm{halobenzene}}+E_{\mathrm{phenol}/\mathrm{indole}}\right)\right)$$

and reported as kJ/mol.

**Figure 7:**
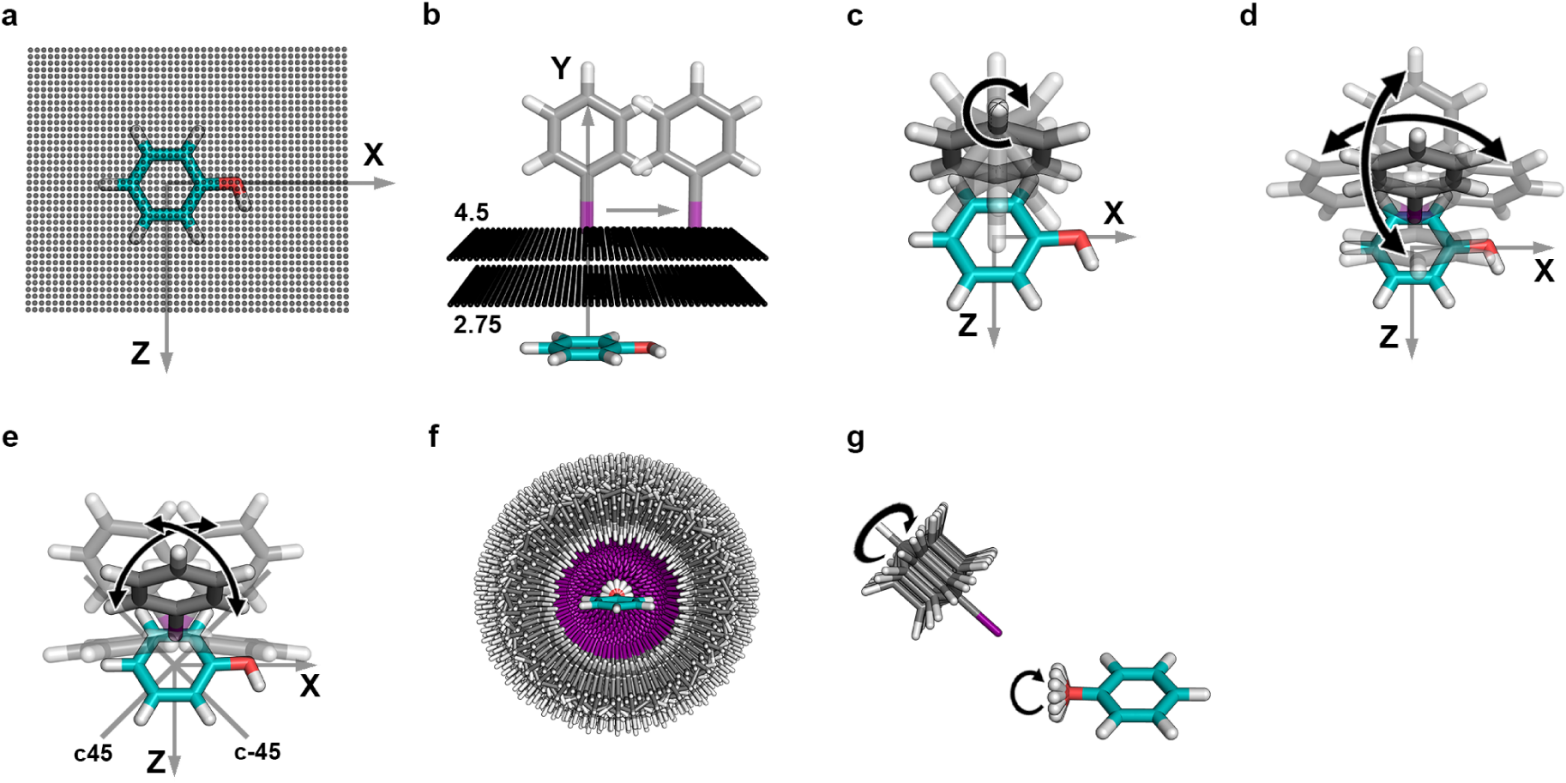
Illustration of the systematic generation of interaction geometries. Grid setups are depicted using phenol as a halogen bond acceptor. (a) Grid points on the XZ-plane were generated with dimensions X_translation_ = [−5.0 Å to 7.0 Å], Z_translation_ = [−5.0 Å to 5.0 Å] in steps of 0.25 Å. (b) Grid points were generated for eight different distances, d_X···π‑plane_ = [2.75 to 4.5 Å] in steps of 0.25 Å, between the halogen atom (Cl, Br, or I) and the phenol plane. (c) Rotations of the halobenzene around the *y*-axis y_rot_ = [0° (initially), 45°, 90°, 135°]. (d) Deviations from the optimal σ-hole angle, α_C‑X···π‑plane_ = 180°, from −40° to 40° in steps of 10°, achieved by rotating around the x- and *z*-axes. (e) Custom-generated rotational axes (c45 and c-45), lying 45° to the x- and *z*-axes. Rotations around these axes are similar to (d). (f) Halobenzenes addressing the phenol’s oxygen atom were generated in a spherical shape around the oxygen with interaction distances d_X···O_ = [2.5–4.5 Å] in steps of 0.25 Å. The C-X vector points toward the oxygen atom, with α_C‑X···O_ = 180°. (g) Rotations of the halobenzene around the C-X axis (similar to (c)) in steps of 30°. Deviations of the phenol’s O–H vector were made in steps of 30°. Figures were prepared with PyMOL.

### Generation of a Random Geometry Training and Test Set

Similar to the generation of the systematic dataset, we generated a smaller subset of 60,000 interaction geometries (20,000 per halobenzene) with random geometric features for each addressed system to test the models’ generalizability. Parameters of X, Z_translation_ = [−5.0 to 7.0 Å], Y_translation_ = [1.5 to 5.0 Å], y_rot_ = [0° to 360°], and α_C‑X···⊥(π‑plane)_ = [0° to 60°] were randomly chosen and applied to a halobenzene. To ensure the uniqueness of the generated geometries, newly chosen parameters were compared to previous ones and only applied if found to be distinct (>0.4 Å between each halogen atom and each neighboring carbon atom of two given molecules) within the dataset. Datasets for retraining (300,000 geometries, 100,000 per halogen) were similarly generated for each model system.

### Feature Extraction and ANN Model Training

All data preprocessing, feature extraction, and learning approaches for all three model systems were built in Python using custom scripts with the *PyTorch* ^68^ and *scikit-learn* packages, two open-source Python libraries for machine learning. ANN models were trained on geometric features extracted from the interaction geometries. The training process for the individual models was performed on the BinAC—bwHPC Cluster.^69^ In general, the feature vectors *v⃗* **=** *(d*_1_, *d*_2_, ..., *d*_*n*_, *a*_1_, ..., *a*_*n*_) comprise individual distance and angle descriptors, with all features detailed in Tables S1 and S2. The adduct formation energy Δ*E* of each geometry serves as the target value. Distance features were computed as all pairwise distances between selected atoms of the halobenzene fragment (the halogen atom, its neighboring carbon, and the two adjacent ring carbons) and every heavy atom of the respective amino acid model system (Figure 8a). Additional distances involving the phenol hydroxyl hydrogen and the imidazole N–H hydrogen were also included.

**Figure 8:**
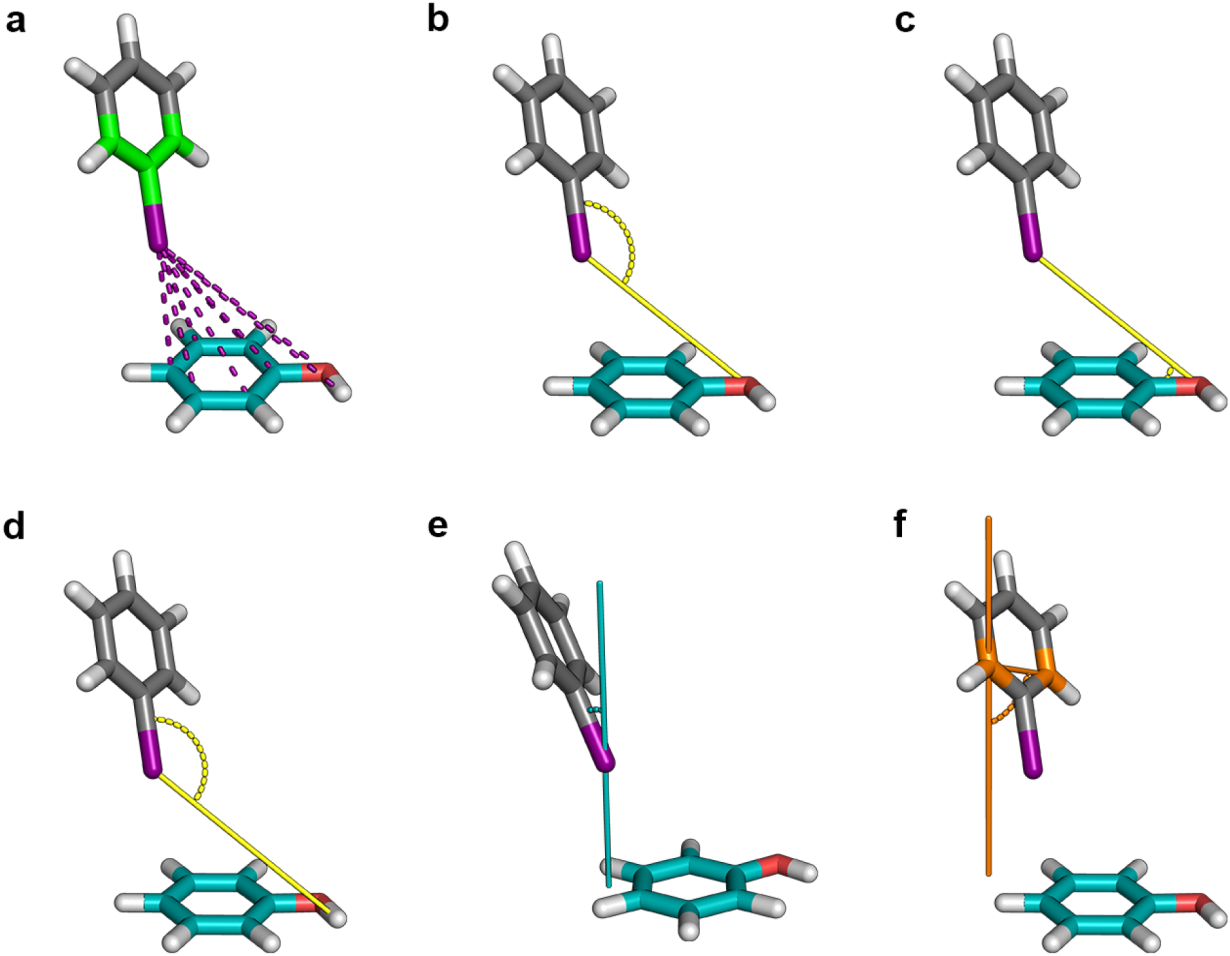
Overview of the feature extraction from halobenzene-phenol interaction geometries. (a) Pairwise distances from the halogen atom and the green-colored carbons of the halobenzene to all heavy atoms of the phenol system, as well as the hydrogen atom of the hydroxyl group. (b) Angle feature of the halogen atom, its neighboring carbon (C-X vector), and the phenol oxygen. (c) Angle feature of the phenol oxygen, its attached hydrogen, and the halogen atom. (d) Angle feature of the hydroxyl hydrogen and the C-X vector. (e) Angle feature of the normal vector of the phenol ring plane and the C-X vector. (f) Angle feature of the vector between the orange-colored carbons of the halobenzene and the normal of the phenol plane.

Angle features were derived from vectors defined between halobenzene and the amino acid model system. For phenol, five angle descriptors were used: (i) the angle between the halogen atom, its neighboring carbon (CX vector), and the phenol oxygen (Figure 8b); (ii) the angle formed by the phenol oxygen, its attached hydrogen, and the halogen atom (Figure 8c); (iii) the angle between the hydroxyl hydrogen and the C-X vector (Figure 8d); (iv) the angle between the normal vector of the phenol ring plane and the C-X vector (Figure 8e); and (v) the angle between the normal vector of the phenol ring plane and the vector defined by the two adjacent halobenzene ring carbons (Figure 8f). The imidazole feature set incorporates four angle descriptors (Figure S5): (i) the angle between the nonprotonated nitrogen and the C-X vector; (ii) the angle between the protonated nitrogen and the C-X vector; (iii) the angle between the normal of the imidazole plane and the C-X vector; and (iv) the angle between the vector defined by the two adjacent halobenzene ring carbons and the normal of the imidazole plane. The indole feature vector contains three angle descriptors (Figure S6): (i) the angle between the C-X vector and the center of mass of the indole heterocycle; (ii) the angle between the indole-plane normal and the C-X vector; and (iii) the angle between the vector defined by the two adjacent halobenzene ring carbons and the indole-plane normal. In total, the phenol, imidazole, and indole feature vectors comprise 37, 28, and 39 distance and angle features, respectively.

Prior to model training, each feature was scaled independently using a min-max normalization procedure (scikit-learn MinMaxScaler). The complete dataset was partitioned into an 80% training portion and a 20% test portion using a stratified 5-fold leave-one-out scheme to ensure reproducibility and balanced sampling (Figure 9). Stratification was performed with respect to (i) the halogen identity and (ii) the halogen···π-plane distance, thereby maintaining comparable feature distributions across all folds. Each training fold was subsequently divided again into a training subset (80%) and an internal validation subset (20%) by using the same stratification criteria.

**Figure 9:**
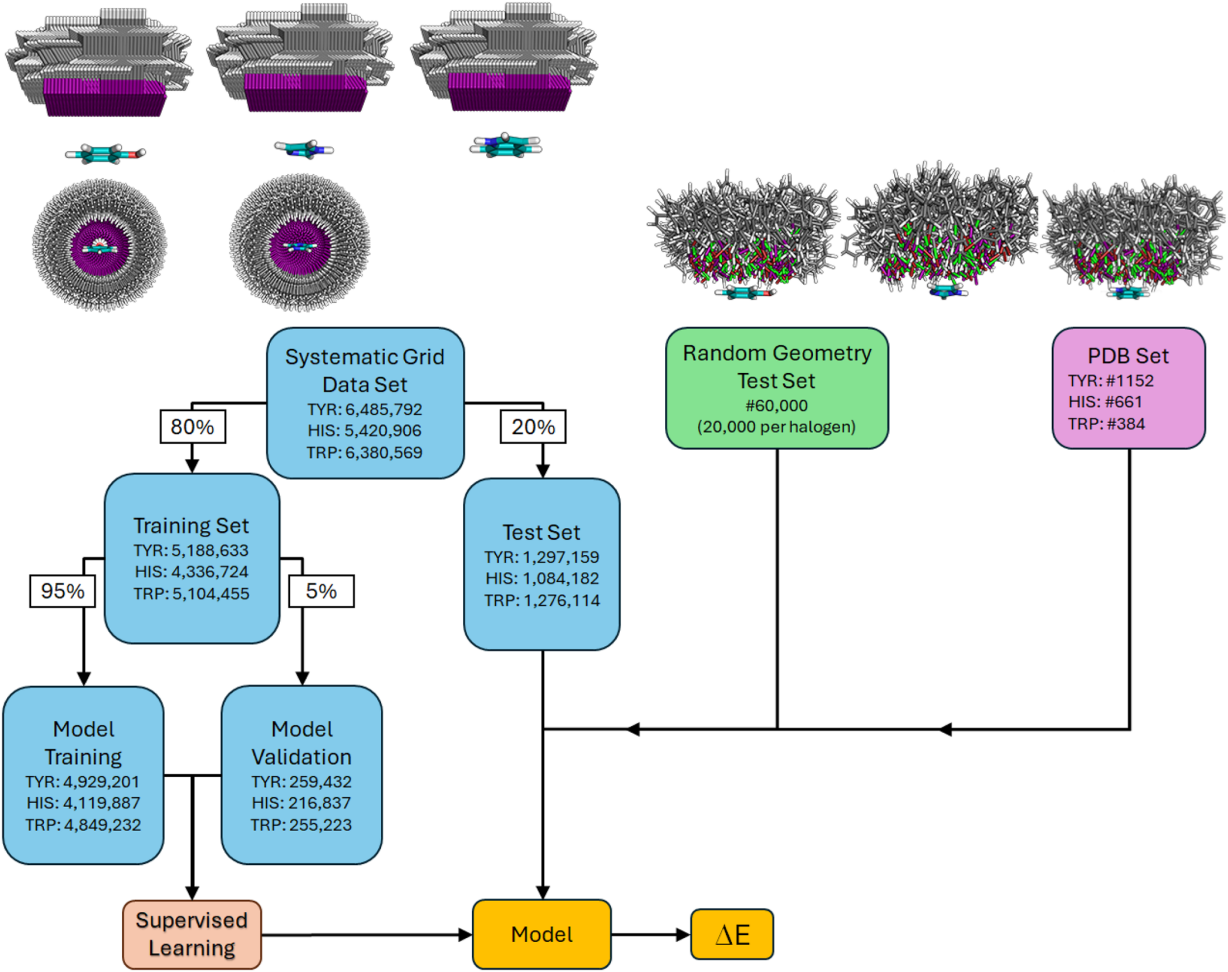
Overview of the different datasets. The systematic grid dataset is split into a training set (80% of the whole dataset) and a test set (20%). The training set is further split into model training (again 80%) and the model validation set (20%). The two model datasets are fed into the model via a supervised training approach. The random geometry test set (60,000 geometries) is used to evaluate the models’ generalized performance on unseen data. The PDB set (N_TYR_ = 1152, N_HIS_ = 661, and N_TRP_ = 384) is used to represent and evaluate biological examples. The respective geometries shown are only a small excerpt of the full datasets.

Model development involves extensive hyperparameter optimization within a supervised learning framework. The search space included different activation functions (Sigmoid, Tanh, and LeakyReLU from PyTorch), network depths of two to four fully connected hidden layers, and a variety of layer-width combinations drawn from [256, 128, 64, 32, 16, and 8]. Additional hyperparameters, including initial learning rate (0.1 to 0.0001), batch size (32 to 2048), and number of training epochs (100 to 10 000), were systematically varied. Training was performed using the Adam optimizer (PyTorch) and an elastic-net-weighted MSE loss to mitigate data imbalance. Early stopping criteria were employed to avoid overfitting. The hyperparameter set that performed best on the validation subset was used for a final training run on a 95%/5% training/validation set split. Model accuracy was assessed using the mean squared error (MSE), root-mean-square error (RMSE), and coefficient of determination (R^2^). High predictive quality is reflected by an R^2^ value approaching 1.0 in combination with low MSE and RMSE values. The RMSE per epoch from the final training phase is presented in Figure S2a,c, and e for each system individually. Energy deviations between computed and predicted adduct formation energies were calculated as in kJ/mol.

2 $$\Delta \Delta E={\Delta E}_{\mathrm{calculated}}-{\Delta E}_{\mathrm{predicted}}$$

The training started with a fixed learning rate and was adapted (the value was halved) down to a minimum of 0.0001 if there had not been a loss improvement for 10 epochs. The final hyperparameter configuration for all three models comprised three fully connected hidden layers with 256, 128, and 64 units, respectively, using Leaky Rectified Linear Unit (Leaky ReLU) activation functions. The initial learning rate was set to 0.001, while batch sizes were set to 1024 for the imidazole model and 2048 for the phenol and indole models.

### PDB Scan of Tyrosine, Histidine, and Tryptophan Acceptors

A PDB (as of July 2025:239, 149) scan was conducted using a custom Python/PyMOL^70^ script. All protein structures were preprocessed by removing alternative conformations, metal ions, and hydrogen atoms. PDB entries were then screened for ligands containing aromatic halogens (Cl, Br, or I) bound to an aromatic ring and comprising at least six heavy atoms. Aromatic amino acid side chain residues of tyrosine, histidine, and tryptophan within 5 Å of the halogen atom were retained as XB acceptors. To increase the chance for plausible XB geometries, an angular criterion was applied, where the C-X bond vector was required to orient toward the π-plane of the residue (α_C‑X···⊥(π‑plane)_ < 50°). A lower distance cutoff of d_X···AA_= 1 Å was employed. The side chain residue of each addressed amino acid was replaced by the respective model system (phenol for tyrosine, imidazole for histidine, and indole for tryptophan). Likewise, the native ligand was substituted by the matching halobenzene (chloro-, bromo-, or iodobenzene), ensuring that the halogen atom position, the C-X bond direction, and the orientation of the aromatic or heteroaromatic plane were preserved by following a matched molecular pair strategy. Both molecular fragments were separately optimized at the MP2/TZVPP level of theory, as described previously. From this procedure, we obtained N_TYR_ = 1152, N_HIS_ = 661, and N_TRP_ = 384 distinct interaction geometries between halobenzene and the respective model system. Single-point calculations were subsequently carried out at the MP2/TZVPP level. Adduct formation energies were computed by using the supermolecular scheme (eq 1).

### Model Evaluation and Outlier Detection

To assess how well the models reproduce adduct formation energies for a given interaction geometry, three independent test sets were evaluated: the 20% withheld test set, the random-geometry test set, and the PDB-derived set. Ideally, these test data represent previously unseen configurations, allowing a meaningful assessment of the models’ generalizability. Model performance was quantified using the root-mean-square error (RMSE) and coefficient of determination (R^2^). Because the random-geometry and PDB test sets may contain structures that fall outside the feature domain sampled during training, we additionally characterized the degree of extrapolation using the Mahalanobis distance (MD). MD provides a multivariate measure of how far a data point lies from the centroid of the training set feature distribution. It is defined as:

3 $$D_{M}\left(x\right)=\sqrt{{\left(x-\mu \right)}^{T}\Sigma^{-1}\left(x-\mu \right)}$$

where *x* denotes the feature vector of the input geometry, *μ* is the mean feature vector of the training data, and Σ is the corresponding covariance matrix. Large MD values indicate that a geometry occupies a low-probability region of feature space relative to the training data, marking it as a potential outlier or extrapolative case. MDs were calculated for all test set geometries, and a threshold was established to identify points classified as outliers.

## Supplementary Material





## Data Availability

PyMOL is an open-source software maintained and distributed by Schrödinger. There is an open-source version of PyMOL available at: https://github.com/schrodinger/pymol-open-source. Python and all of its’ packages are an open-source programming language available and downloadable from https://www.python.org/. PyTorch is an open-source machine learning library for Python: https://pytorch.org/. TURBOMOLE is a purchasable software maintained and distributed by *TURBOMOLE GmbH*. Demo versions are available at https://www.turbomole.org/. The licensed software was provided to us by the bwHPC Cluster JUSTUS2.

## Notes

Graphical depiction of distance- and angle-based features; detailed description of the features used for training; results of the training and validation process of final models; histograms of energy differences and random geometry and PDB-derived test sets; graphical depiction of grid generation and feature extraction for imidazole and indole datasets; summary of model performances; and learning curve analysis (PDF)
Detailed tables of the individual data points of the random geometry dataset and the PDB dataset with corresponding adduct formation energies for calculated and predicted energies (XLSX)

## Abbreviations

XB: halogen bond
X: halogen
QM: quantum mechanical
MP2: Møller–Plesset perturbation method order 2
TZVPP: valence triple-ζ with two sets of polarization functions
SCF: self-consistent field
RI: resolution of identity
RMSE: root-mean-square error
ECP: effective core potential
NN: neural network
R^2^: coefficient of determination
MD: Mahalanobis Distance
PDB: Protein Data Bank
PHE: phenylalanine
TYR: tyrosine
HIS: histidine
TRP: tryptophan
MSE: mean squared error
CCSD­(T): coupled cluster with single, double, and perturbative triple excitations

## References

[1] Bissantz C., Kuhn B., Stahl M. (2010). A Medicinal Chemist’s Guide
to Molecular Interactions. J. Med. Chem..

[2] Adhav V. A., Saikrishnan K. (2023). The Realm
of Unconventional Noncovalent Interactions
in Proteins: Their Significance in Structure and Function. ACS Omega.

[3] Anighoro A. (2020). Underappreciated
Chemical Interactions in Protein–Ligand Complexes. Quantum Mech. Drug Discovery.

[4] Jena S., Dutta J., Tulsiyan K. D., Sahu A. K., Choudhury S. S., Biswal H. S. (2022). Noncovalent interactions
in proteins and nucleic acids:
Beyond hydrogen bonding and π-stacking. Chem. Soc. Rev..

[5] Müller-Dethlefs K., Hobza P. (2000). Noncovalent
Interactions: A Challenge for Experiment and Theory. Chem. Rev..

[6] Clark T., Hennemann M., Murray J. S., Politzer P. (2007). Halogen bonding: The
σ-hole. J. Mol. Model..

[7] Desiraju G. R., Ho P. S., Kloo L., Legon A. C., Marquardt R., Metrangolo P., Politzer P., Resnati G., Rissanen K. (2013). Definition
of the halogen bond (IUPAC Recommendations 2013). Pure Appl. Chem..

[8] Politzer P., Murray J. S., Clark T. (2010). Halogen bonding:
An electrostatically-driven
highly directional noncovalent interaction. Phys. Chem. Chem. Phys..

[9] Wang C., Danovich D., Mo Y., Shaik S. (2014). On The Nature of the
Halogen Bond. J. Chem. Theory Comput..

[10] Cavallo G., Metrangolo P., Milani R., Pilati T., Priimagi A., Resnati G., Terraneo G. (2016). The Halogen Bond. Chem. Rev..

[11] Heidrich J., Exner T. E., Boeckler F. M. (2019). Predicting the Magnitude of σ-Holes
Using VmaxPred, a Fast and Efficient Tool Supporting the Application
of Halogen Bonds in Drug Discovery. J. Chem.
Inf. Model..

[12] Bhattarai S., Sutradhar D., Chandra A. K. (2021). Tuning of halogen-bond
strength:
Comparative role of basicity and strength of σ-hole. J. Mol. Struct..

[13] Donald K. J., Pham N., Ravichandran P. (2023). Sigma Hole Potentials as Tools: Quantifying
and Partitioning Substituent Effects. J. Phys.
Chem. A.

[14] Esrafili M. D., Mahdavinia G., Javaheri M., Sobhi H. R. (2014). A theoretical
study
of substitution effects on halogen−π interactions. Mol. Phys..

[15] Lange A., Heidrich J., Zimmermann M. O., Exner T. E., Boeckler F. M. (2019). Scaffold
Effects on Halogen Bonding Strength. J. Chem.
Inf. Model..

[16] Sedlak R., Kolář M.
H., Hobza P. (2015). Polar Flattening
and
the Strength of Halogen Bonding. J. Chem. Theory
Comput..

[17] Berger G., Frangville P., Meyer F. (2020). Halogen bonding for
molecular recognition:
New developments in materials and biological sciences. Chem. Commun..

[18] Erdélyi M. (2012). Halogen bonding
in solution. Chem. Soc. Rev..

[19] Hardegger L. A., Kuhn B., Spinnler B., Anselm L., Ecabert R., Stihle M., Gsell B., Thoma R., Diez J., Benz J. (2011). Systematic
Investigation of Halogen Bonding in Protein–Ligand
Interactions. Angew. Chem., Int. Ed..

[20] Hernandes Z. M., Cavalcanti T. S. M., Moreira M. D. R., de Azevedo Junior F. W., Leite L. A. C. (2010). Halogen Atoms in the Modern Medicinal Chemistry: Hints
for the Drug Design. Curr. Drug Targets.

[21] Parker A. J., Stewart J., Donald K. J., Parish C. A. (2012). Halogen Bonding
in DNA Base Pairs. J. Am. Chem. Soc..

[22] Scholfield M. R., Zanden C. M. V., Carter M., Ho P. S. (2013). Halogen bonding
(X-bonding): A biological perspective. Protein
Sci..

[23] Shinada N. K., de Brevern A. G., Schmidtke P. (2019). Halogens in Protein–Ligand
Binding Mechanism: A Structural Perspective. J. Med. Chem..

[24] Sirimulla S., Bailey J. B., Vegesna R., Narayan M. (2013). Halogen Interactions
in Protein–Ligand Complexes: Implications of Halogen Bonding
for Rational Drug Design. J. Chem. Inf. Model..

[25] Xu Z., Yang Z., Liu Y., Lu Y., Chen K., Zhu W. (2014). Halogen Bond Its Role beyond Drug–Target
Binding Affinity
for Drug Discovery and Development. J. Chem.
Inf. Model..

[26] Walker M. G., Mendez C. G., Ho A. N., Czarny R. S., Rappé A. K., Ho P. S. (2025). Design of a halogen bond catalyzed DNA endonuclease. Proc. Natl. Acad. Sci. U. S. A..

[27] Wilcken R., Zimmermann M. O., Bauer M. R., Rutherford T. J., Fersht A. R., Joerger A. C., Boeckler F. M. (2015). Experimental and
Theoretical Evaluation of the Ethynyl Moiety as a Halogen Bioisostere. ACS Chem. Biol..

[28] Dammann M., Stahlecker J., Zimmermann M. O., Klett T., Rotzinger K., Kramer M., Coles M., Stehle T., Boeckler F. M. (2022). Screening
of a Halogen-Enriched Fragment Library Leads to Unconventional Binding
Modes. J. Med. Chem..

[29] Vaas S., Zimmermann M. O., Schollmeyer D., Stahlecker J., Engelhardt M. U., Rheinganz J., Drotleff B., Olfert M., Lämmerhofer M., Kramer M. (2023). Principles and Applications
of CF2X Moieties as Unconventional Halogen Bond Donors in Medicinal
Chemistry, Chemical Biology, and Drug Discovery. J. Med. Chem..

[30] Jiang L., Zhang X., Zhou Y., Chen Y., Luo Z., Li J., Yuan C., Huang M. (2018). Halogen bonding for the design of
inhibitors by targeting the S1 pocket of serine proteases. RSC Adv..

[31] Lu Y., Liu Y., Li H., Zhu X., Liu H., Zhu W. (2012). Energetic
Effects between Halogen Bonds and Anion-π or Lone Pair-π
Interactions: A Theoretical Study. J. Phys.
Chem. A.

[32] Matter H., Nazaré M., Güssregen S., Will D. W., Schreuder H., Bauer A., Urmann M., Ritter K., Wagner M., Wehner V. (2009). Evidence for C-Cl/C-Br···π Interactions
as an Important Contribution to Protein–Ligand Binding Affinity. Angew. Chem., Int. Ed..

[33] Parisini E., Metrangolo P., Pilati T., Resnati G., Terraneo G. (2011). Halogen bonding
in halocarbon–protein complexes: A structural survey. Chem. Soc. Rev..

[34] Politzer P., Murray J. S., Clark T. (2013). Halogen bonding
and other σ-hole
interactions: A perspective. Phys. Chem. Chem.
Phys..

[35] Rowe R. K., Ho P. S. (2017). Relationships between hydrogen bonds and halogen bonds in biological
systems. Acta Crystallogr., Sect. B.

[36] Ford M. C., Ho P. S. (2016). Computational Tools
To Model Halogen Bonds in Medicinal Chemistry. J. Med. Chem..

[37] Young, D. C. Computational Chemistry: A Practical Guide for Applying Techniques to Real World Problems; Wiley, 2001. DOI: 10.1002/0471220655.

[38] Zhu Z., Xu Z., Zhu W. (2020). Interaction Nature and Computational Methods for Halogen
Bonding: A Perspective. J. Chem. Inf. Model..

[39] Wilcken R., Zimmermann M. O., Lange A., Zahn S., Boeckler F. M. (2012). Using halogen
bonds to address the protein backbone: A systematic evaluation. J. Comput. Aided Mol. Des..

[40] Wilcken R., Zimmermann M. O., Lange A., Joerger A. C., Boeckler F. M. (2013). Principles
and Applications of Halogen Bonding in Medicinal Chemistry and Chemical
Biology. J. Med. Chem..

[41] Zimmermann M. O., Boeckler F. M. (2016). Targeting the protein
backbone with aryl halides: Systematic
comparison of halogen bonding and π···π
interactions using N-methylacetamide. Med. Chem.
Commun..

[42] Wilcken R., Zimmermann M. O., Lange A., Zahn S., Kirchner B., Boeckler F. M. (2011). Addressing Methionine in Molecular Design through Directed
Sulfur–Halogen Bonds. J. Chem. Theory
Comput..

[43] Lange A., Zimmermann M. O., Wilcken R., Zahn S., Boeckler F. M. (2013). Targeting
Histidine Side Chains in Molecular Design through Nitrogen–Halogen
Bonds. J. Chem. Inf. Model..

[44] Zimmermann M. O., Lange A., Zahn S., Exner T. E., Boeckler F. M. (2016). Using Surface
Scans for the Evaluation of Halogen Bonds toward the Side Chains of
Aspartate, Asparagine, Glutamate, and Glutamine. J. Chem. Inf. Model..

[45] Engelhardt M. U., Zimmermann M. O., Dammann M., Stahlecker J., Poso A., Kronenberger T., Kunick C., Stehle T., Boeckler F. M. (2024). Halogen Bonding
on Water—A Drop in the Ocean?. J. Chem.
Theory Comput..

[46] Zhou P., Lv J., Zou J., Tian F., Shang Z. (2010). Halogen–water–hydrogen
bridges in biomolecules. J. Struct Biol..

[47] Engelhardt M. U., Zimmermann M. O., Mier F., Boeckler F. M. (2025). Comparison of QM
Methods for the Evaluation of Halogen−π Interactions
for Large-Scale Data Generation. J. Chem. Theory
Comput..

[48] Engelhardt M. U., Mier F., Zimmermann M. O., Boeckler F. M. (2025). A QM-AI Approach
for the Acceleration of Accurate Assessments of Halogen-π Interactions
by Training Neural Networks. J. Chem. Inf. Model..

[49] Berman H. M., Westbrook J., Feng Z., Gilliland G., Bhat T. N., Weissig H., Shindyalov I. N., Bourne P. E. (2000). The Protein Data Bank. Nucleic
Acids Res..

[50] Mahalanobis, P. C. On the generalized distance in statistics Proceedings of the National Institute of Sciences of India National Institute of Sciences of India 1936 12 49–55

[51] Perez M. A. S., Bassani-Sternberg M., Coukos G., Gfeller D., Zoete V. (2019). Analysis of Secondary
Structure Biases in Naturally Presented HLA-I
Ligands. Front. Immunol..

[52] Devore D. P., Shuford K. L. (2024). Data and Molecular Fingerprint-Driven Machine Learning
Approaches to Halogen Bonding. J. Chem. Inf.
Model..

[53] Anstine D. M., Zubatyuk R., Isayev O. (2025). AIMNet2: A neural network
potential
to meet your neutral, charged, organic, and elemental-organic needs. Chem. Sci..

[54] Head-Gordon M., Pople J. A., Frisch M. J. (1988). MP2 energy evaluation by direct methods. Chem. Phys. Lett..

[55] Møller C., Plesset M. S. (1934). Note on an Approximation
Treatment for Many-Electron
Systems. Phys. Rev..

[56] TURBOMOLE V7.7.1 2019; A development of University of Karlsruhe and Forschungszentrum Karlsruhe GmbH, 1989–2007, TURBOMOLE GmbH, 2007.

[57] Schäfer A., Huber C., Ahlrichs R. (1994). Fully optimized contracted Gaussian
basis sets of triple zeta valence quality for atoms Li to Kr. J. Chem. Phys..

[58] bwForCluster - JUSTUS 2; https://wiki.bwhpc.de/e/JUSTUS2.

[59] Feyereisen M., Fitzgerald G., Komornicki A. (1993). Use of approximate integrals in ab
initio theory. An application in MP2 energy calculations. Chem. Phys. Lett..

[60] Häser M., Ahlrichs R. (1989). Improvements on the
direct SCF method. J. Comput. Chem..

[61] Hättig C. (2003). Geometry optimizations
with the coupled-cluster model CC2 using the resolution-of-the-identity
approximation. J. Chem. Phys..

[62] Hättig C., Hellweg A., Köhn A. (2006). Distributed memory parallel implementation
of energies and gradients for second-order Møller–Plesset
perturbation theory with the resolution-of-the-identity approximation. Phys. Chem. Chem. Phys..

[63] Hättig C., Weigend F. (2000). CC2 excitation energy
calculations on large molecules
using the resolution of the identity approximation. J. Chem. Phys..

[64] Hoffmann R. (1963). An Extended
Hückel Theory. I. Hydrocarbons. J. Chem.
Phys..

[65] Weigend F., Ahlrichs R. (2005). Balanced basis sets
of split valence, triple zeta valence
and quadruple zeta valence quality for H to Rn: Design and assessment
of accuracy. Phys. Chem. Chem. Phys..

[66] Weigend F., Häser M. (1997). RI-MP2: First
derivatives and global consistency. Theor. Chem.
Acc..

[67] Weigend F., Häser M., Patzelt H., Ahlrichs R. (1998). RI-MP2: Optimized auxiliary
basis sets and demonstration of efficiency. Chem. Phys. Lett..

[68] PyTorch; https://pytorch.org/.

[69] bwForCluster - BinAC; https://wiki.bwhpc.de/e/BinAC.

[70] The PyMOL Molecular Graphics System, Version 3.1; Schrödinger, LLC, 2015.

