# Supplementary material for: Expanding and Enhancing Neural Network-Based QM-AI Models for the Accurate Prediction of Halogen‑π Interaction Energies in Protein Contexts
Source: J Chem Inf Model. 2026 Apr 14;66(8):4409–26. doi: 10.1021/acs.jcim.5c03249 (PMC13126628; doi:10.1021/acs.jcim.5c03249)
Supplement: Supplementary file 1 [file ci5c03249_si_001.pdf]

Supporting Information

Expanding and Enhancing Neural Network-Based QM-AI Models for the Accurate Prediction of Halogen-π Interaction Energies in Protein Contexts

Marc U. Engelhardt[1], Finn Mier[1], Markus O. Zimmermann[1,2], Frank M. Boeckler[1,2,]*

[1] Laboratory for Molecular Design & Pharmaceutical Biophysics, Institute of Pharmaceutical Sciences, Department of Pharmacy and Biochemistry, Eberhard Karls Universität Tübingen, 72076 Tübingen, Germany.

[2] Interfaculty Institute for Biomedical Informatics (IBMI), Eberhard Karls Universität Tübingen, 72076 Tübingen, Germany.

Corresponding Author

*Frank M. Boeckler: frank.boeckler@uni-tuebingen.de

This file contains the following information:

Supplementary Figures

- Supplementary Figure S1: Depiction of distance-based features and the atom-naming convention for phenol, imidazole, and indole in complex with iodobenzene
- Supplementary Figure S2: Results of the training and validation process for the final phenol, imidazole, and indole models.
- Supplementary Figure S3: Histograms of energy differences illustrating the model performance on the random geometry test sets of the three models for phenol, imidazole, and indole
- Supplementary Figure S4: Histograms of energy differences illustrating the model performance on the PDB-derived test sets (tyrosine, histidine, tryptophan set) of the three models for phenol, imidazole, and indole
- Supplementary Figure S5: Grid generation and feature extraction of halobenzene-imidazole interaction geometries.
- Supplementary Figure S6: Grid generation and feature extraction of halobenzene-indole interaction geometries.
- Supplementary Figure S7: Learning curve analysis with different training set sizes.

Supplementary Tables:

- Supplementary Table S1: Overview of the phenol features derived from interaction geometries
- Supplementary Table S2: Overview of the imidazole and indole features derived from corresponding interaction geometries
- Supplementary Table S3: Summary of performance for all models in terms of R^2 and RMSE (kJ/mol).

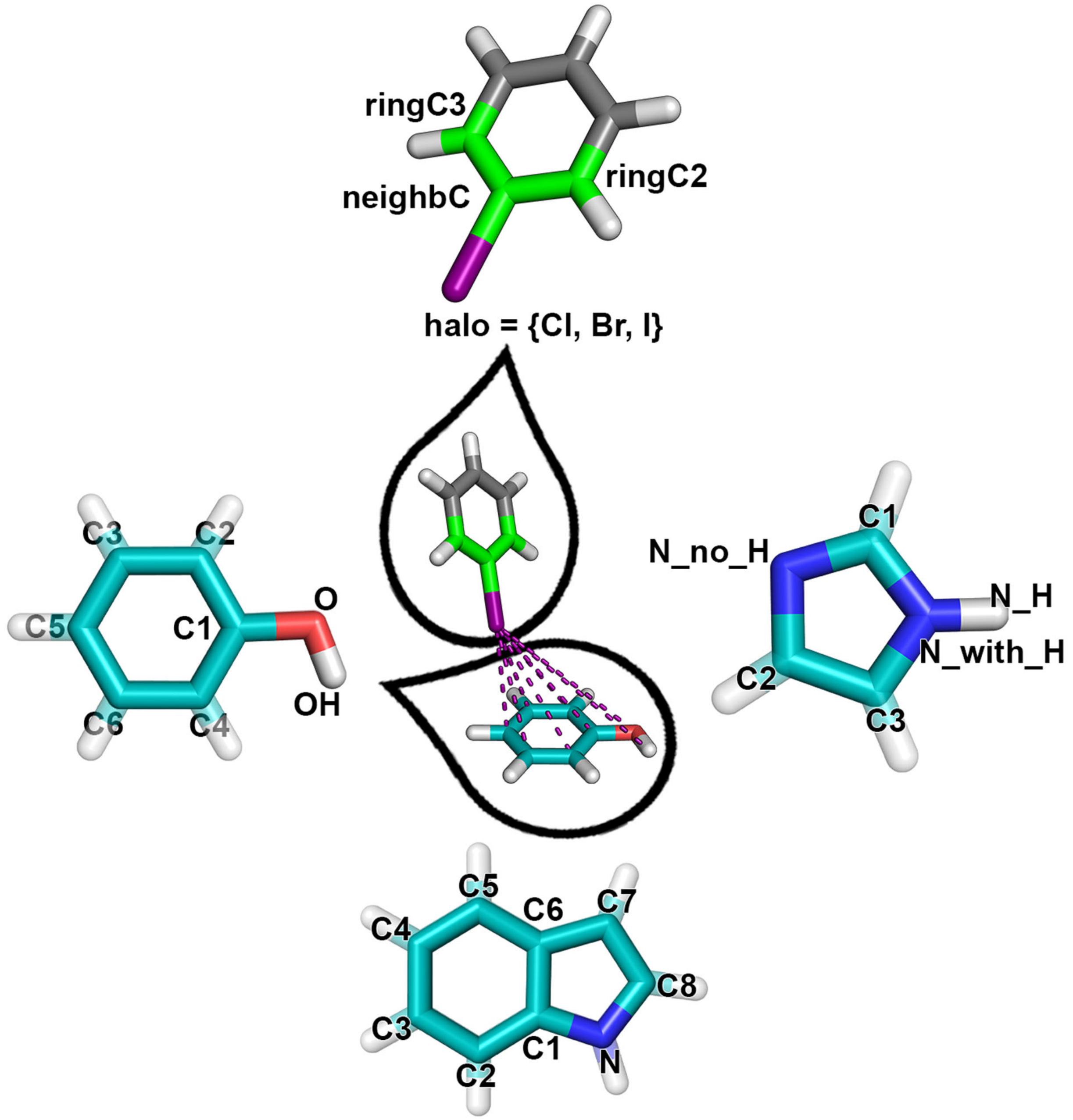

Figure S1. Depiction of distance-based features and the corresponding atom-naming conventions for phenol (left), imidazole (right), indole (bottom), and halobenzene (top, shown here as iodobenzene). The central illustration highlights the pairwise distances between the halogen and each feature atom of the phenol system. In the detailed views, phenol carbon atoms are labeled counterclockwise starting from the carbon atom attached to the oxygen. In halobenzene, the carbon

directly bonded to the halogen is labeled neighbC, while ringC2 and ringC3 denote the nearest carbons in the aromatic ring to that neighbor. Pairwise distance features are similarly generated for the imidazole and indole system.

Table S1: Overview of the features derived from the phenol interaction geometries. Feature names follow the atom-naming scheme shown in Figure S4. Distances are reported in Å, and angles in degrees. The final three features are Boolean values indicating the presence of the respective halogen.

Phenol Feature Name	Description
O_halo	Distance halogen to the oxygen of the hydroxyl group
O_neighbC	Distance neighboring carbon of the halogen to the oxygen of the hydroxyl group
O_ringC2	Distance of ring carbon C2 (ringC2) of the halobenzene to the oxygen of the hydroxyl group
O_ringC3	Distance of ring carbon C3 (ringC3) of the halobenzene to the oxygen of the hydroxyl group
OH_halo	Distance halogen to the hydrogen atom of the hydroxyl group
OH_neighbC	Distance neighboring carbon of the halogen to the hydrogen of the hydroxyl group
OH_ringC2	Distance of ring carbon C2 (ringC2) of the halobenzene to the hydrogen of the hydroxyl group
OH_ringC3	Distance of ring carbon C3 (ringC3) of the halobenzene to the hydrogen of the hydroxyl group
C1_halo	Distance halogen to the first phenol ring carbon atom C1 adjacent to the hydroxyl's oxygen
C1_neighbC	Distance halogen neighbor to phenol C1

C1_ringC2	Distance ringC2 to phenol C1
C1_ringC3	Distance ringC3 to phenol C1
C2_halo	Distance halogen to the second phenol ring carbon atom C2
C2_neighbC	Distance halogen neighbor to phenol C2
C2_ringC2	Distance ringC2 to phenol C2
C2_ringC3	Distance ringC3 to phenol C2
C3_halo	Distance halogen to phenol ring carbon atom C3
C3_neighbC	Distance halogen neighbor to phenol C3
C3_ringC2	Distance ringC2 to phenol C3
C3_ringC3	Distance ringC3 to phenol C3
C4_halo	Distance halogen to phenol ring carbon atom C4
C4_neighbC	Distance halogen neighbor to phenol C4
C4_ringC2	Distance ringC2 to phenol C4
C4_ringC3	Distance ringC3 to phenol C4
C5_halo	Distance halogen to phenol ring carbon atom C5
C5_neighbC	Distance halogen neighbor to phenol C5
C5_ringC2	Distance ringC2 to phenol C5
C5_ringC3	Distance ringC3 to phenol C5
C6_halo	Distance halogen to phenol ring carbon atom C6
C6_neighbC	Distance halogen neighbor to phenol C6
C6_ringC2	Distance ringC2 to phenol C6
C6_ringC3	Distance ringC3 to phenol C6
Angle1_F-O_L-X_L-C	Angle between the oxygen atom of the phenol, the halogen atom, and the neighboring carbon atom
Angle2_F-OH_F-O_L-X	Angle between the hydrogen atom of the hydroxyl group, the oxygen atom, and the halogen atom
Angle3_F-OH_L-X_L-C	Angle between the hydrogen atom of the hydroxyl group, the halogen atom, and the neighboring carbon atom

Angle4_normal_phe_plane_L-X_L-C	Angle between the C-X vector (of neighbC and the halogen) and the normal of the phenol plane
Angle5_phe-plane_L_rC2-rC3	Angle between the ringC2-ringC3 vector and the normal of the phenol plane
is_Cl	1 if X = Cl, else 0
is_Br	1 if X = Br, else 0
is_I	1 if X = I, else 0

Table S2: Overview of the features derived from the imidazole and indole interaction geometries. Feature names follow the atom-naming scheme shown in Figure S4. Distances are reported in Å, and angles in degrees. The final three features are Boolean values indicating the presence of the respective halogen. Description of the features is similar to table S1.

Imidazole Feature Name	Indole Feature Name
N_no_H_halo	N_halo
N_no_H_neighbC	N_neighbC
N_no_H_ringC2	N_ringC2
N_no_H_ringC3	N_ringC3
N_with_H_halo	C1_halo
N_with_H_neighbC	C1_neighbC
N_with_H_ringC2	C1_ringC2
N_with_H_ringC3	C1_ringC3
N_H_halo	C2_halo
N_H_neighbC	C2_neighbC

N_H_ringC2	C2_ringC2
N_H_ringC3	C2_ringC3
C1_halo	C3_halo
C1_neighbC	C3_neighbC
C1_ringC2	C3_ringC2
C1_ringC3	C3_ringC3
C2_halo	C4_halo
C2_neighbC	C4_neighbC
C2_ringC2	C4_ringC2
C2_ringC3	C4_ringC3
C3_halo	C5_halo
C3_neighbC	C5_neighbC
C3_ringC2	C5_ringC2
C3_ringC3	C5_ringC3
Angle1_F-N_no_H_L-X_L-C	C6_halo
Angle2_F-N_with_H_L-X_L-C	C6_neighbC
Angle3_imi-plane_L-X_L-C	C6_ringC2
Angle4_imi-plane_L-X_neighb_C_ring_C1_2	C6_ringC3
is_Cl	C7_halo
is_Br	C7_neighbC
is_I	C7_ringC2
	C7_ringC3
	C8_halo
	C8_neighbC
	C8_ringC2
	C8_ringC3

Angle1_CoM_L-X_L-C

Angle2_indol-plane_L-X_L-C

Angle3_indol-plane_L_rC2-rC3

is_Cl

is_Br

is_I

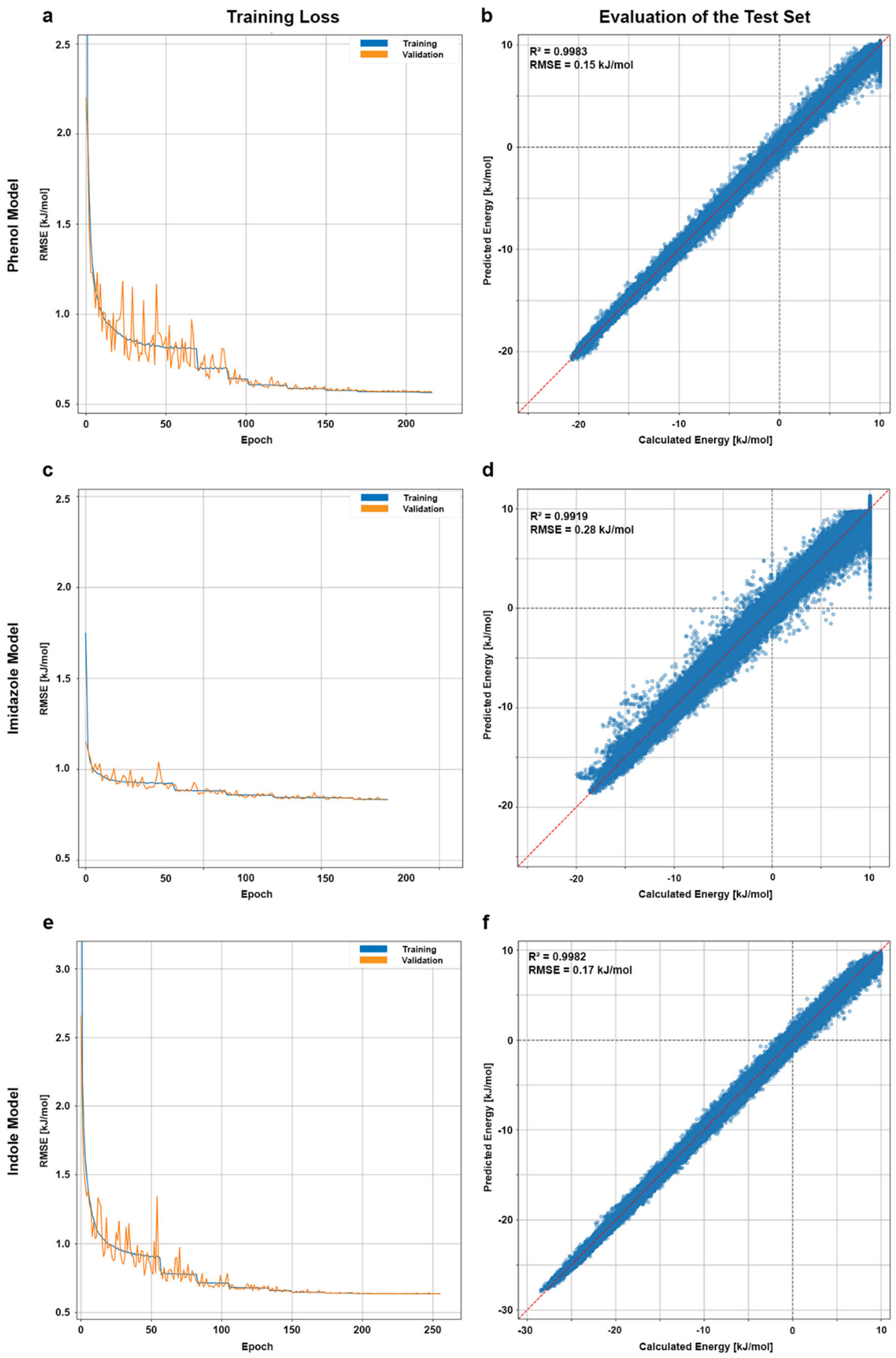
a
Training Loss
b
Evaluation of the Test Set
Phenol Model
RMSE [kJ/mol]
Epoch
Training
Validation
R² = 0.9983
RMSE = 0.15 kJ/mol
Predicted Energy [kJ/mol]
Calculated Energy [kJ/mol]
c
d
Imidazole Model
R² = 0.9919
RMSE = 0.28 kJ/mol
e
f
Indole Model
R² = 0.9982
RMSE = 0.17 kJ/mol

64

Figure S2. Results of the training process for the final phenol, imidazole, and indole models. Panels (a), (c), and (e) show the root-mean-square error (RMSE, kJ/mol) as a function of training epoch. Blue curves correspond to the training RMSE for each epoch, while orange curves show the validation RMSE evaluated after each epoch. Panels (b), (d), and (f) display the final model performance on the validation set, with coefficients of determination of $R_{phenol\text{-}val}^2$ = 0.9983, $R_{imidazole\text{-}val}^2$ = 0.9919, $R_{indole\text{-}val}^2$ = 0.9982, and corresponding RMSE values of 0.15, 0.28 kJ/mol, and 0.17 kJ/mol, respectively. Calculated adduct formation energies are plotted against the predicted values. The red dashed line denotes perfect agreement between calculated and predicted energies, while the gray dashed lines indicate the transition between negative and positive energies.

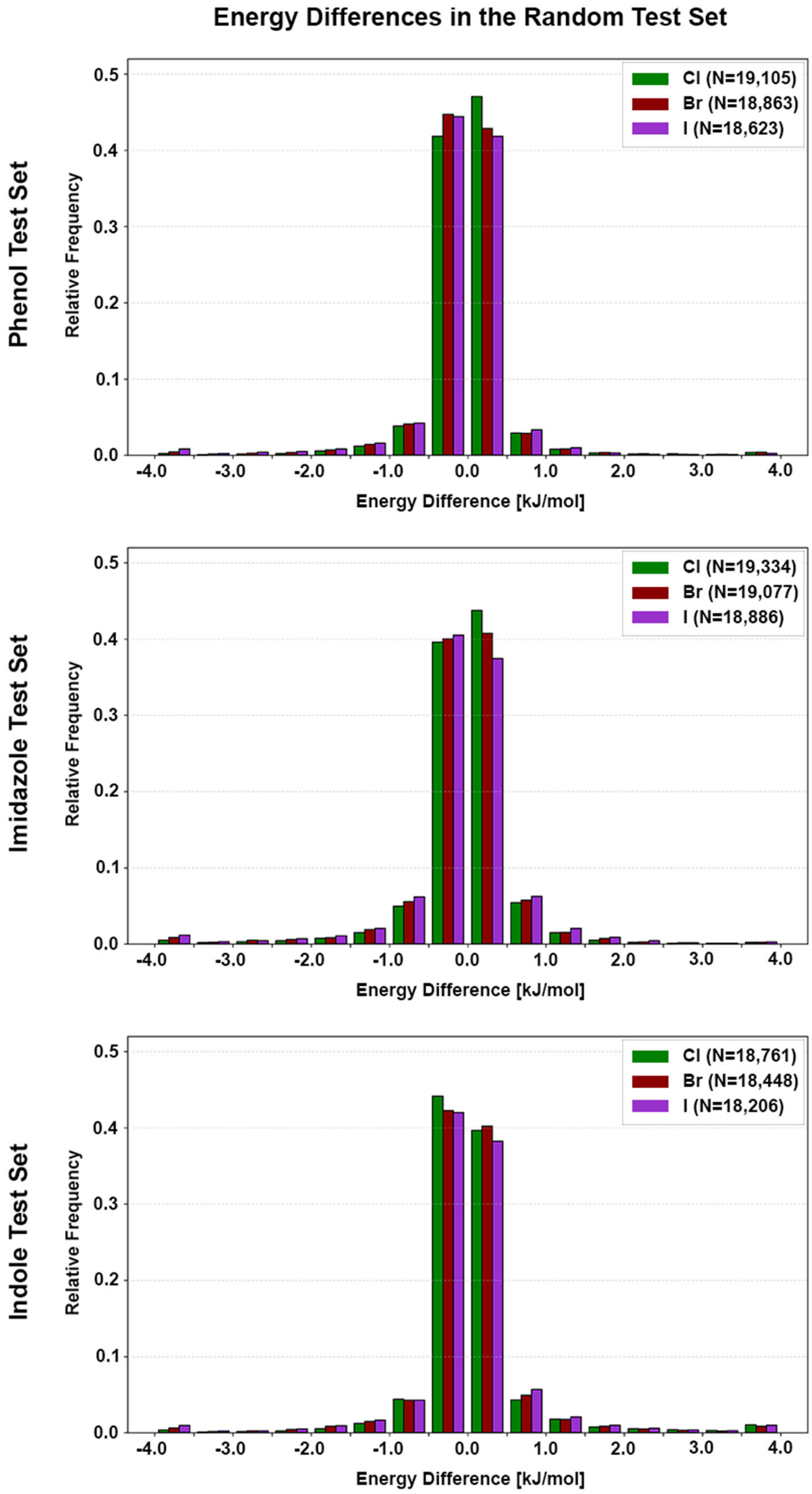

Energy Differences in the Random Test Set
Phenol Test Set
Relative Frequency
Cl (N=19,105)
Br (N=18,863)
I (N=18,623)
Energy Difference [kJ/mol]
Imidazole Test Set
Relative Frequency
Cl (N=19,334)
Br (N=19,077)
I (N=18,886)
Energy Difference [kJ/mol]
Indole Test Set
Relative Frequency
Cl (N=18,761)
Br (N=18,448)
I (N=18,206)
Energy Difference [kJ/mol]

Figure S3. Model performance on the random geometry test sets of the three models for phenol, imidazole, and indole. Histograms show the relative frequencies of energy differences between calculated and predicted energy in bins of 0.5 kJ/mol from -4.0 kJ/mol to 4.0 kJ/mol for chlorine (green), bromine (dark red), and iodine (purple) separately. Larger values are clipped to the respective limitation for better visibility.

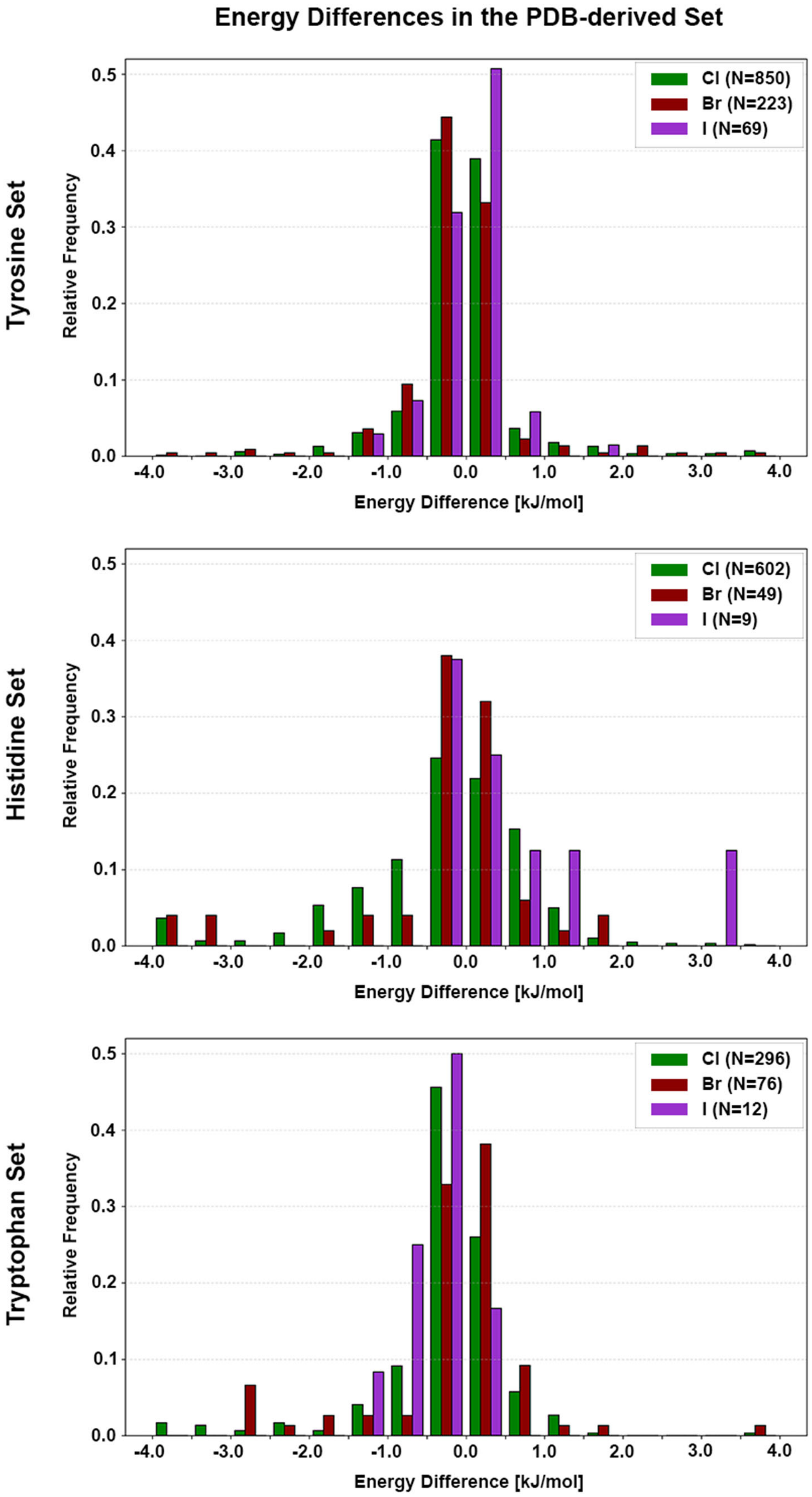
Energy Differences in the PDB-derived Set
Tyrosine Set
Relative Frequency
Cl (N=850)
Br (N=223)
I (N=69)
Energy Difference [kJ/mol]
Histidine Set
Relative Frequency
Cl (N=602)
Br (N=49)
I (N=9)
Energy Difference [kJ/mol]
Tryptophan Set
Relative Frequency
Cl (N=296)
Br (N=76)
I (N=12)
Energy Difference [kJ/mol]

86

Figure S4. Model performance on the PDB-derived test sets (tyrosine, histidine, and indole set) of the three models for phenol, imidazole, and indole. Histograms show the relative frequencies of energy differences between calculated and predicted energy in bins of 0.5 kJ/mol from -4.0 kJ/mol to 4.0 kJ/mol for chlorine (green), bromine (dark red), and iodine (purple) separately. Larger values are clipped to the respective limitation for better visibility.

Table S3: Summary of performance for all models in terms of R^2 and RMSE (kJ/mol). Performance is reported for the initial training evaluation and for external validation on random-geometry and PDB-derived datasets. External evaluations were performed either on subsets (step (i)) or on the complete data sets (step (ii)), for both initial and enhanced model variants.

model	evaluation set	evaluation step	R^2	RMSE [kJ/mol]
phenol	initial training evaluation	- [a]	0.9983	0.15
	random geometry set	subset, step (i)	0.9935	0.27
		complete set, step (ii)	0.9644	0.90
	PDB-derived set	subset, step (i)	0.9904	0.30
		complete set, step (ii)	0.9530	0.76
phenol enhanced	initial training evaluation	- [a]	0.9958	0.34
	random geometry set	complete set, step (ii)	0.9851	0.58
	PDB-derived set	complete set, step (ii)	0.9610	0.69
imidazole	initial training evaluation	- [a]	0.9919	0.28
	random geometry set	subset, step (i)	0.9848	0.34
		complete set, step (ii)	0.9096	0.94
	PDB-derived set	subset, step (i)	0.9834	0.43

		complete set, step (ii)	0.8840	1.65
imidazole enhanced	initial training evaluation	- [a]	0.9827	0.45
	random geometry set	complete set, step (ii)	0.9555	0.86
	PDB-derived set	complete set, step (ii)	0.9252	1.35
indole	initial training evaluation	- [a]	0.9982	0.17
	random geometry set	subset, step (i)	0.9953	0.28
		complete set, step (ii)	0.9269	1.17
	PDB-derived set	subset, step (i)	0.9900	0.33
		complete set, step (ii)	0.8819	1.33
indole enhanced	initial training evaluation	- [a]	0.9952	0.39
	random geometry set	complete set, step (ii)	0.9803	0.80
	PDB-derived set	complete set, step (ii)	0.9179	1.11

[a] No further subdivision into step (i) and step (ii) was performed for the training evaluation. Instead, a single withheld test set was used, which was generated by stratified partitioning of the full training dataset.

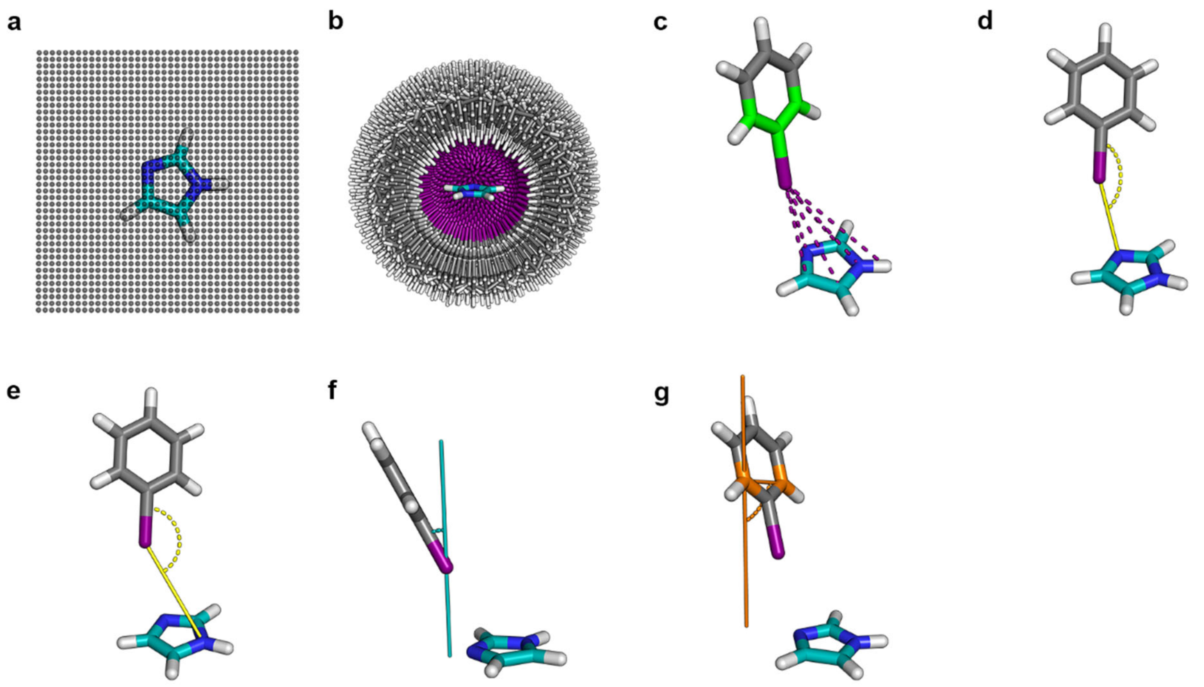

Figure S5. Illustration of the systematic generation of interaction geometries between halobenzenes and imidazole, as well as the feature extraction. (a) Grid points on the XZ-plane were generated with dimensions $X,Z_{translation}$ = [-5.0 Å to 5.0 Å] in steps of 0.25 Å and in eight different distances, $d_{X\cdots\pi\text{-plane}}$ = [2.75 Å to 4.5 Å] in steps of 0.25 Å, between the halogen atom (Cl, Br, or I) and the imidazole plane (similar to the description in the manuscript). (b) Additional geometries addressing the deprotonated nitrogen atom of the imidazole system in a hemi-spherical shape (c) Pairwise distances from the halogen atom and the green colored carbons of the halobenzene to all heavy atoms and the imidazole N-H hydrogen. (d) Angle feature of the halogen atom, its neighboring carbon and the deprotonated nitrogen of the imidazole. (e) Angle feature of the halogen atom, its neighboring carbon and the protonated nitrogen of the imidazole. (f) Angle feature of the C-X vector and the normal of the imidazole plane $\alpha_{C\text{-}X\cdots\perp(\pi\text{-plane})}$. (g) Angle feature of the vector between the orange-colored carbons of the halobenzene and the normal of the imidazole plane. Figures were prepared with PyMOL.

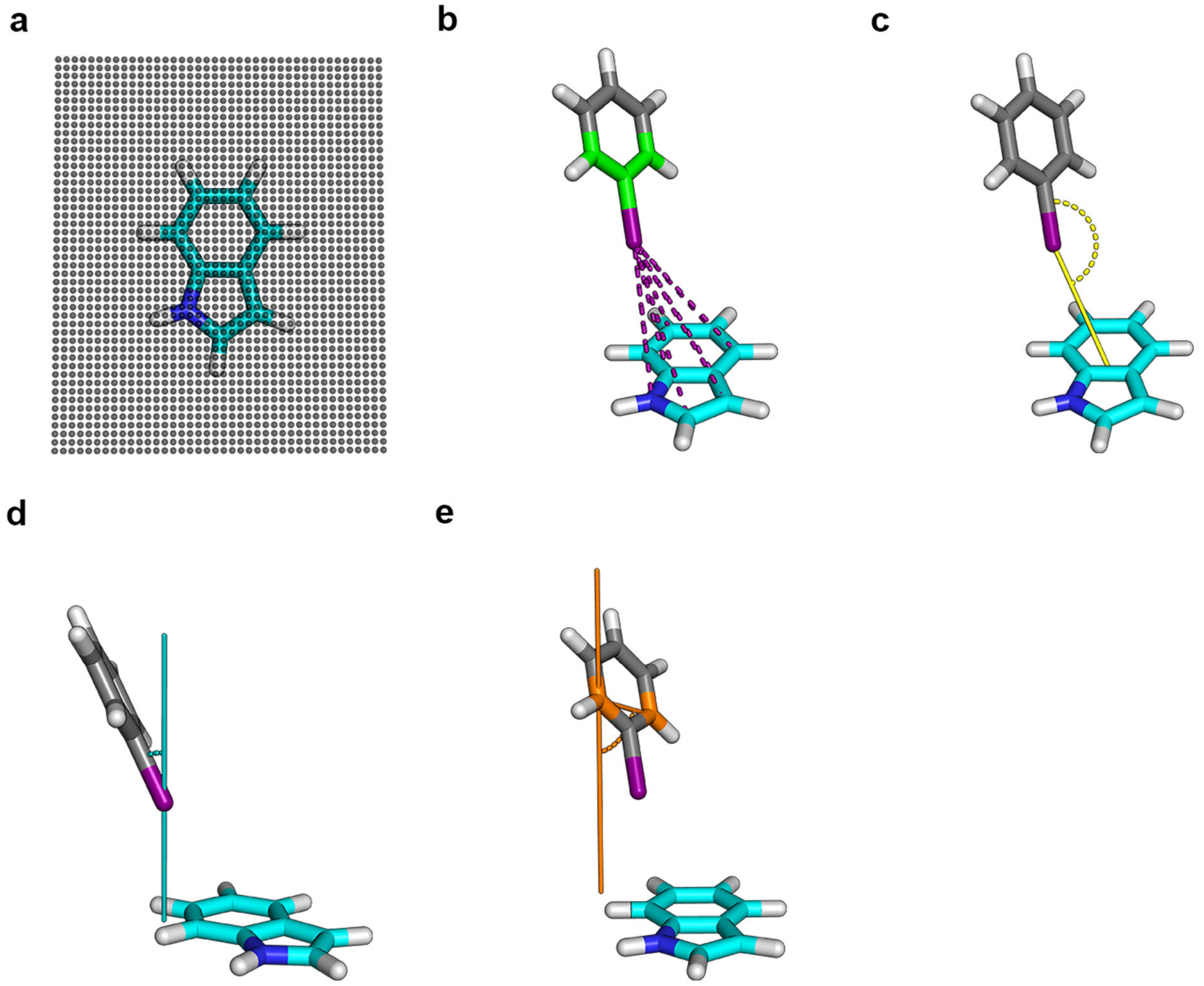

Figure S6. Illustration of the systematic generation of interaction geometries between halobenzenes and indole, as well as the feature extraction. (a) Grid points on the XZ-plane were generated with dimensions $X_{translation}$ = [-5.0 Å to 5.0 Å], $Z_{translation}$ = [-5.0 Å to 7.0 Å] in steps of 0.25 Å and in eight different distances, $d_{X\cdots\pi\text{-plane}}$ = [2.75 Å to 4.5 Å] in steps of 0.25 Å, between the halogen atom (Cl, Br, or I) and the imidazole plane (similar to the description in the manuscript). (b) Pairwise distances from the halogen atom and the green colored carbons of the halobenzene to all heavy atoms of the indole system. (c) Angle feature of the halogen atom, its neighboring carbon and centroid of the indole. (d) Angle feature of the C-X vector and the normal

of the indole plane $\alpha_{C\text{-}X\cdots\perp(\pi\text{-plane})}$. (g) Angle feature of the vector between the orange-colored carbons of the halobenzene and the normal of the indole plane. Figures were prepared with PyMOL.

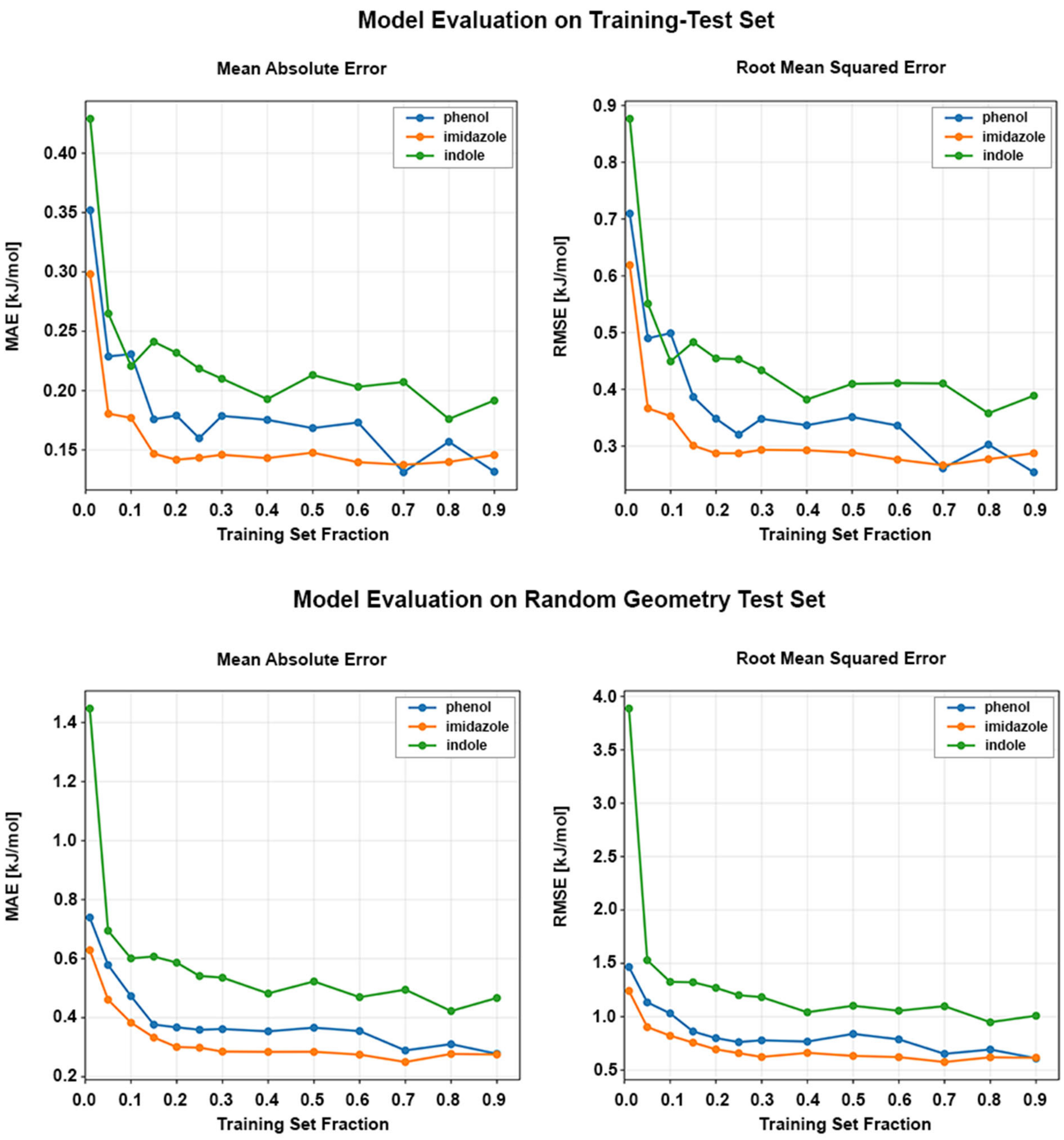

Figure S7. Learning curve analysis of the three models with different training set sizes given in fractions of the whole dataset. Evaluation was performed on the complementary test set fraction, as well as on the random geometry test set. Performance is reported as mean-absolute-error (MAE) and root-mean-squared-error (RMSE) in kJ/mol.
